# Cascade loop of ferroptosis induction and immunotherapy based on metal‐phenolic networks for combined therapy of colorectal cancer

**DOI:** 10.1002/EXP.20230117

**Published:** 2024-05-15

**Authors:** Yuwei Li, Yuxi Duan, Yunyi Li, Yuan Gu, Lu Zhou, Zhongting Xiao, Xinying Yu, Yanjun Cai, Erzhuo Cheng, Qianqian Liu, Yong Jiang, Quan Yang, Feng Zhang, Qi Lei, Bin Yang

**Affiliations:** ^1^ School of Biomedical Engineering The Fourth Affiliated Hospital of Guangzhou Medical University Guangzhou Medical University Guangzhou People's Republic of China; ^2^ Department of Nephrology First Affiliated Hospital of Jinan University Guangzhou People's Republic of China; ^3^ Provincial Key Laboratory of Allergy and Clinical Immunology The Second Affiliated Hospital Guangzhou Medical University Guangzhou People's Republic of China

**Keywords:** colorectal cancer, ferroptosis, immunotherapy

## Abstract

Cancer immunotherapy is the most promising method for tumor therapy, while ferroptosis could activate the immunogenicity of cancer and strengthen the cellular immune response. However, limited by the complex tumor microenvironment, the abundant glutathione (GSH) and low reactive oxygen species (ROS) seriously weaken ferroptosis and the immune response. Herein, the authors report photothermal metal‐phenolic networks (MPNs) supplied with buthionine sulfoximine (BSO) by reducing levels of GSH and then trapping the tumor cells in the ferroptosis and immunotherapy cascade loop to eliminate colorectal cancer (CRC). The MPNs coated with the model antigen ovalbumin can accumulate at the tumor site, mediate immunogenic cell death (ICD) under NIR irradiation, and initiate tumoricidal immunity. Then the activated CD8^+^ T cells would release IFN‐γ to inhibit GPX4 and promote the immunogenic ferroptosis induced by Fe^3+^ and BSO. Finally, the tumor cells at intertumoral and intratumoral levels would be involved in the ferroptosis‐dominated cancer‐immunity circle for CRC eradication, resulting in outstanding therapeutic outcomes in both primary and distant tumor models. Overall, this strategy employs a photothermal nanoplatform to rapidly stimulate ICD and restrain the oxidation defense system, which provides a promising approach to significantly amplify the “cascade loop” of ferroptosis induction and immunotherapy for treatment of CRC.

## Graphical abstract

Herein, a photothermal nanoplatform based on ferric ions (Fe^3+^), naturally‐derived polyphenol gallic acid (GA), model antigen ovalbumin (OVA), and l‐buthionine sulfoximine (BSO) was constructed, which can supply with GPX4 inhibitors to eliminate colorectal cancer (CRC) by trapping the tumor cells in the ferroptosis and immunotherapy cascade loop. This strategy provides a promising approach to significantly amplify ferroptosis induction and immunotherapy for treatment of CRC.

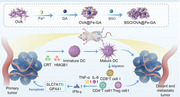

## 1 INTRODUCTION

Colorectal cancer (CRC) featured with genomic instability and inflammatory tumor microenvironment (TME) is a highly aggressive malignancy, which ranks third in mortality of all cancers due to the high clinical misdiagnosis rate (≈30%) at early stage, the high liver metastatic rate (over 50%) at end‐stage, and inherent low response rate to current surgery, radiotherapy, and chemotherapy.^[^ ^1^ ^]^ Immunotherapy is a burgeoning treatment strategy that fights CRC by boosting the inherent and acquired immune defense.^[^ ^2^ ^]^ Unfortunately, the therapeutic outcome is compromised by the high heterogeneity and resistance of CRC, which disfavors the systemic antitumor immunity to eradicate the primary foci and inhibit tumor metastasis/recurrence.^[^ ^3^ ^]^ Therefore, it is necessary to study effective treatment strategies to steadily induce immune reaction to the heterogeneous cancer cells with distinctive antigen expression at the intertumoral and intratumoral levels, thus extending the life expectancy.

Ferroptosis, a non‐apoptotic regulated cell lethal mechanism proposed in 2012, is predominantly induced by a disequilibrium between iron, lipid hydroperoxides, and intracellular antioxidant systems.^[^ ^4^ ^]^ Being different from caspase‐dependent apoptosis pathway that could be suppressed resulting from the intrinsic or acquired apoptotic resistance of CRC cells,^[^ ^5^ ^]^ ferroptosis exhibits great therapeutic potential for the therapy‐resistant CRC subgroups with rat sarcoma gene (RAS)‐mutation due to their elevated oxidative stress levels.^[^ ^6^ ^]^ Meanwhile, ferroptotic cancer cells would release damage‐associated molecular pattern (DAMP) signals by high‐mobility group box protein 1 (HMGB1) emission and calreticulin (CRT) translocation onto the surface, resulting in the easy recruiting of dendritic cells (DCs) to process the tumor‐associated antigens (TAAs) and initiate the cytotoxic T lymphocyte (CD8^+^ T cell) for anticancer immunity.^[^ ^7^ ^]^ On the other hand, recent researches have revealed that interferon gamma (IFN‐γ) released by CD8^+^ T cells in tumoricidal immunity can downregulate the expression of solute carrier 7A11 (SLC7A11) and solute carrier 3A2 (SLC3A2), two subunits of the glutamate‐cystine antiporter system x_c_^−^, impair the uptake of cystine, reduce the synthesis of glutathione (GSH), and suppress the activity of glutathione peroxidase 4 (GPX4), thus facilitating lipid peroxidation and ferroptosis induction.^[^ ^8^ ^]^ Theoretically, such a cascade loop of ferroptosis induction and immunotherapy would not stop among tumor cells until ferroptosis is blockaded. We believe that it would be a tailored treatment for heterogeneous CRC.

**SCHEME 1:**
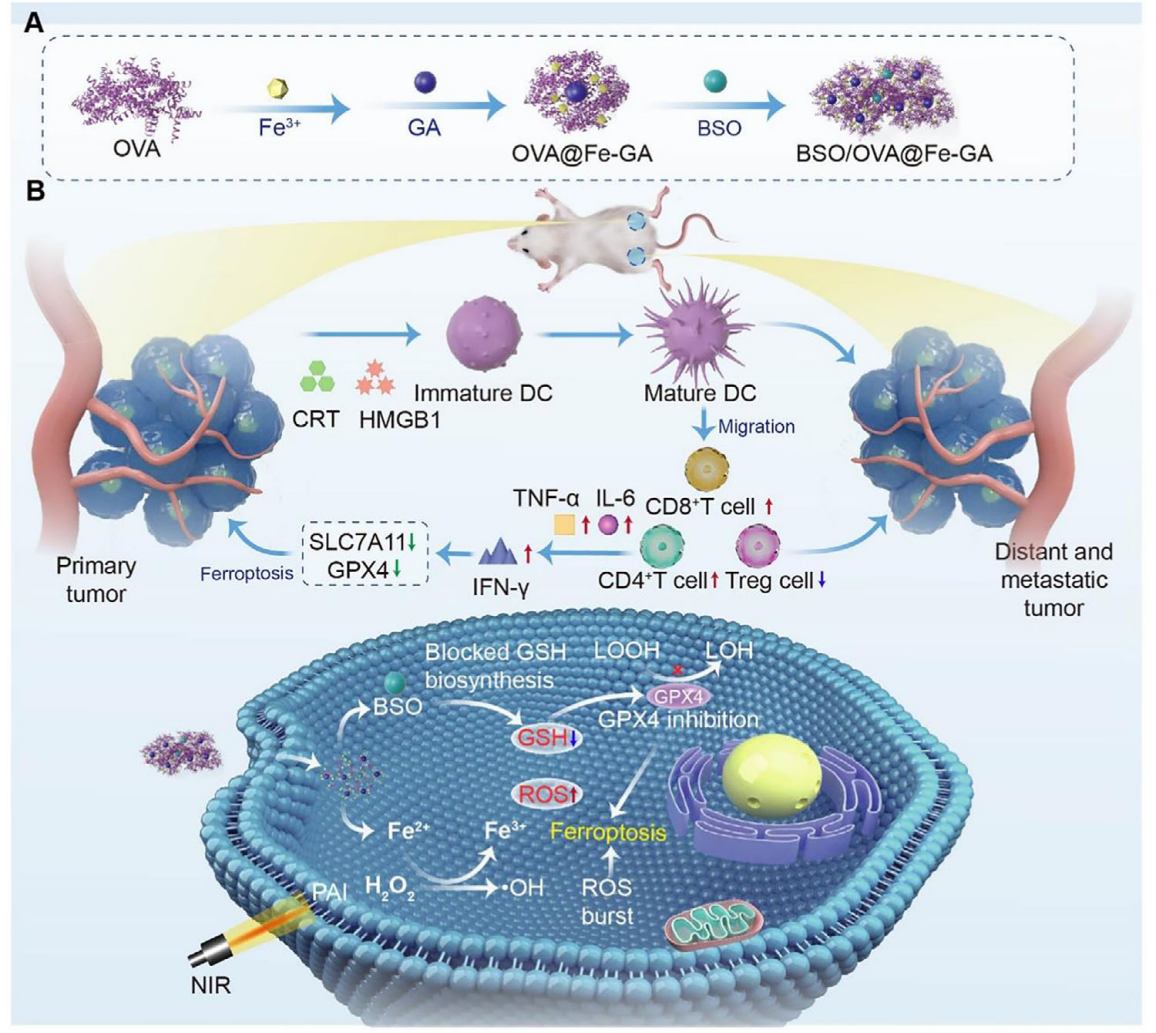
Ferroptosis mediated cascade loop strategy based on metal‐phenolic networks for photothermal/immune therapy of colorectal cancer. (A) Schematic of the synthesis of BOFG. (B) Schematic illustration of BOFG for PTT‐enhanced cyclically amplified tumor ferroptosis‐immunotherapy.

A variety of cellular metabolic pathways and antioxidant systems protect CRC cells from ferroptosis,^[^ ^9^ ^]^ and wherein GPX4 employing glutathione (GSH) as the substrate plays the central role in avoiding lipid peroxidation and evading ferroptotic cell death.^[^ ^10^ ^]^ GSH depletion using intracellular Fenton reaction or GSH synthesis inhibition using l‐buthionine sulfoximine (BSO) are preferable tactics for GPX4 inhibition.^[^ ^11^ ^]^ Herein, we construct a photothermal nanoplatform supplied with GPX4 inhibitors to mediate the cascade loop of ferroptosis induction and immunotherapy of CRC. Specifically, MPNs with photothermal conversion effect,^[^ ^12^ ^]^ formed by ferric ions (Fe^3+^) and naturally‐derived polyphenol gallic acid (GA), were coated on the BSO and model antigen of ovalbumin (OVA) to construct BSO/OVA@Fe‐GA (BOFG) (Scheme 1). Antigen‐shielded BOFG with the diameter less than 75 nm could preferentially accumulate at the tumor site depending on the advantage of the enhanced permeability and retention (EPR) effect.^[^ ^13^ ^]^ The photothermal effect under NIR irradiation, the Fenton reaction catalyzed by Fe^3+^‐GA MPNs to generate hydroxyl radical and the BSO‐blocked GSH synthesis inactivating GPX4 collectively facilitate BOFG to induce ICD of CRC. Then, the released TAAs would promote DCs antigen‐presentation and maturation, and thus boost T cells proliferation and infiltration.^[^ ^14^ ^]^ Subsequently CD8^+^ T cells release IFN‐γ to inhibit neighboring tumor cells uptaking cystine for GSH synthesis, which further enhance the suppression of GPX4 and sensitize the cell to immunogenic ferroptosis inducing by Fe^3+^ and BSO. Finally, the tumor cells at intertumoral and intratumoral levels would be involved in the ferroptosis‐dominated cancer‐immunity circle for complete CRC ablation. The in vitro and in vivo results have suggested the fine therapeutic outcome mediated by BOFG under NIR irradiation. Overall, the strategy simultaneously initiating ICD and suppressing the oxidation defense system significantly amplify the “cascade loop” of ferroptosis induction and immunotherapy for treatment of drug resistant CRC.

## 2 EXPERIMENTAL SECTION

### 2.1 Materials

FeCl_3_·6H_2_O and Albumin from chicken egg white were purchased from Aladdin (Shanghai, China). Gallic acid was obtained from Damao Chemical (Tianjin, China). SDS‐PAGE, Quickblock, l‐Buthionine‐sulfoximine (BSO) and 2′,7′‐Dichlorofluorescin diacetate (DCFH‐DA) were purchased from Sigma‐Aldrich (Saint Louis, USA). Glutathione (GSH) test kit, mitochondrial membrane potential assay kit with JC‐1 and 4,6‐diamidino‐2‐phenylindole (DAPI) were purchased from Beyotime (Shanghai, China). BODIPY^581/591^‐C11 was purchased from Thermo Fisher (Waltham, MA). Fetal bovine serum (FBS), was purchased from Biological Industries Israel Beit Haemek (Israel). 3‐(4,5‐dimethylth‐iazol‐2‐yl)‐2,5‐diphenyltetrazolium bromide (MTT) was purchased from Adamas‐beta (Shanghai, China). Methylene blue (MB) was purchased from Tianxin (Tianjin, China). Ferrostatin‐1 (Fer‐1) was purchased from Targetmol (Shanghai, China). Calcein‐AM/PI was bought from Solarbio Science and Technology (Beijing, China). FITC anti‐mouse CD3, APC anti‐mouse CD4, PE anti‐mouse CD8a, Brilliant Violet 605 anti‐mouse CD86, PE/Cy7 anti‐mouse CD80, PerCP/Cy5.5 anti‐mouse CD11b, PE anti‐mouse Foxp3, FITC anti‐mouse CD25 were purchased from BioLegend (Beijing, China). Mouse IFN‐γ Elisa kit, Mouse TNF‐α Elisa kit, and Mouse IL‐6 Elisa kit were purchased from Dakewe (Shenzhen, China). Anti‐Glutathione Peroxidase 4 antibody, anti‐CRT antibody, and anti‐HMGB1 were purchased from Abcam (Cambridge, UK).

### 2.2 Synthesis of BSO/OVA@Fe‐GA

Firstly, 45 mg of OVA powder was dissolved utilizing 20 mL of ultrapure H_2_O. Then 54.1 µL of FeCl_3_ (100 mg mL^−1^) was dropwise added into OVA solution under vigorous stirring. After 30 min of stirring, 1.02 mL of gallic acid (10 mg mL^−1^) was dropwise added into OVA@ Fe solution under vigorous stirring and the solution was incubating in dark environment for 2 h, then the OVA@Fe‐GA NPs were purified via centrifugation at 8000 rpm. To fabricate BSO/OVA@Fe‐GA nanoparticles, 4 mg of BSO was dropwise added into OVA@Fe‐GA solution at the Fe^3+^ concentration of 3.48 mm under vigorous stirring. The obtained nanoparticles were collected via centrifugation at 5000 rpm.

### 2.3 Characterizations

The morphologies of nanoparticles were observed by using JEM‐1400PLUS transmission electron microscope (TEM) (Tokyo, Japan) operating at an accelerating voltage of 120 kV. ZetasizerNano ZS equipment (Malvern, UK) was utilized to measure dynamic light scattering (DLS) and zeta potential. The photothermal conversion performance was assessed by LSR‐PS‐IIS 808 nm NIR laser (Zhejiang, China) and recorded by FOTRIC 222s infrared thermal camera (Beijing, China). Zeiss Axio Observer 7 inverted fluorescence microscope (Oberkochen, Germany) or Zeiss LSM980 confocal laser scanning microscope (CLSM) (Oberkochen, Germany) to acquire the fluorescent images. The absorbance of 96‐well plates was tested by an Epoch microplatereader (Vermont, USA). The flow cytometry analyses were taken with CytoFLEX (California, USA). The Western blot of image was acquired with Amersham Imager 680 (California, USA). Photoacoustic performance was investigated by a multi‐modal imaging platform, Vevo LAZR‐X (Tokyo, Japan).

### 2.4 Iron ion release study

BSO/OVA@Fe‐GA was suspended in HAc‐NaAc buffer with pH of 5.0 and stirred at room temperature for release test. Then 1 mL suspension was taken at 30 min, 240 min, 600 min and were reacted with 1 mL of l‐ascorbic acid solution (20 mm). After stirring for 12 h, 0.1% (w/v) 1,10‐phenanthroline solution (2 mL) was added and stirred overnight. Finally, the absorbance at 512 nm was measured by UV–Vis spectrophotometer, and the release amount of iron ion was calculated according to the standard curve.

### 2.5 Methylene blue degradation

To investigate the rate of Fenton‐reaction, a methylene blue (MB) degradation experiment was carried out HAc‐NaAc buffer containing 10 mm H_2_O_2_, 10 µg mL^−1^ MB and 100 µm BSO/OVA@Fe‐GA was incubated at 37°C under mild shaking for 2 h. Then the solutions were filtered by a 220‐nm membrane and the variation of absorbance at 660 nm was measured by UV–Vis spectroscopy.

### 2.6 Photothermal effect study

To assess the photothermal properties of BSO/OVA@Fe‐GA, 1 mL of BSO/OVA@Fe‐GA water suspensions at different concentrations were added to a cuvette and irradiated by laser irradiator. Specifically, 1 mL of BSO/OVA@Fe‐GA (1 mm) was added into a cuvette and irradiated by laser with 1 W cm^−2^ output density. Then the system was placed at RT for a 10‐min cooling period.

### 2.7 In vitro cytotoxicity

MTT assay was conducted to investigate the cytotoxicity in CT26 cells. Cells were seeded in 96‐well plates and cultured with 5% CO_2_ at 37°C overnight. Then the culture medium was replaced by DMEM containing free BSO, OVA@Fe‐GA, and BSO/OVA@Fe‐GA at different concentrations and cultured for 24 h. To test the cytotoxicity of BSO/OVA@Fe‐GA + NIR, the cells were irradiated with laser at a density of 1 W cm^−2^ after incubation for 8 h. After another 16‐h incubation, the medium was replaced by MTT and cultured for 4 h. Finally, the medium was replaced by DMSO and the absorbance was measured by microplatereader.

### 2.8 Live/dead cell staining

In brief, CT26 cells were incubated with PBS, OVA@Fe‐GA, BSO/OVA@Fe‐GA, and BSO/OVA@Fe‐GA + NIR. After 24 h and washed by PBS, the cells were stained using Calcein‐AM/PI according to the instruction, and then inverted fluorescence microscopy was used to observe the intracellular fluorescence.

### 2.9 ROS detection and LPO generation

Intracellular ROS levels were measured by the assay kit with DCFH‐DA. Briefly, CT26 cells were seeded in confocal dish and cultured in DMEM overnight. Then the culture medium was replaced by DMEM containing different samples (PBS, BSO, OVA@Fe‐GA, BSO/OVA@Fe‐GA). After 8‐h incubation, the medium was replaced by DMEM containing 10 µm DCFH‐DA and cultured for 30 min. Then the cells were washed for two times with PBS for CLSM observation and FCM detection.

Intracellular LPO levels were determined via the BODIPY^581/591^‐C11 fluorescent probe (Ex: 485 nm, Em: 520 nm) with green fluorescence. CT26 cells were seeded in dishes at a density of 5 × 10^5^ cells per well and cultured in DMEM overnight. Then the culture medium was replaced by DMEM containing different samples (PBS, BSO, OVA@Fe‐GA, BSO/OVA@Fe‐GA). After 8 h of incubation, the medium was replaced by DMEM containing 5 µm BODIPY^581/591^‐C11 and cultured for 30 min. Then the cells were washed for two times with PBS for CLSM observation.

JC‐1 probe was used to investigate the changes in ΔΨm. For fluorescence imaging, CT26 cells were treated with PBS, BSO, OVA@Fe‐GA, BSO/OVA@Fe‐GA, respectively. After 8 h of incubation, the medium was replaced by 1 × JC‐1 working solution and cultured for 20 min. Then the cells were washed for CLSM observation. For FCM, CT26 cells were seeded in 12‐well plate at a density of 5 × 10^5^ cells per well and cultured overnight. Then the culture medium was replaced by DMEM containing different samples. After 8 h of incubation, the medium was replaced by 1 × JC‐1 working solution and cultured for 20 min. Then the cells were digested and washed for two times with PBS for FCM.

### 2.10 GSH assay and GPX4 detection

The cellular GSH levels were evaluated using GSH and GSSG assay kit. CT26 cells (5 × 10^5^/well) were seeded in a 6‐well plate for 24 h culture firstly. Next, cells were treated with medium containing different samples (PBS, BSO, OVA@Fe‐GA, BSO/OVA@Fe‐GA). After 24 h incubation, the cells were collected and examined following the protocol of GSH assay kit.

To investigate the expression of GPX4, CT26 cells were seeded in 6‐well plate and cultured at 37°C overnight. Then the culture medium was replaced by DMEM containing different samples (PBS, BSO, OVA@Fe‐GA, BSO/OVA@Fe‐GA). After 24 h, the cells were collected and lysed in 100 µL RIPA buffer on ice, then was centrifuged at 10,000 × *g* for 5 min. The supernatant was collected and the concentration of total protein was quantified by BCA assay. Samples were heated with loading buffer at 95°C and proteins were separated by SDS‐PAGE with 20 µg of total protein loaded and transferred to a PVDF membrane and blocked by Quickblock for 15 min and rinsed by TBST for three times. Then the membrane was incubated in anti‐GPX4 primary antibody solution for 2 h followed by rinsing the membrane with TBST for three times and incubating with secondary antibody solution for 1 h with constant shaking. Finally, the membrane was rinsed with TBST for three times and the image was acquired with Amersham Imager 680. The band intensity was calculated using Image Studio.

### 2.11 In vitro immunogenic cell death (ICD) effect

CT26 cells were cultured overnight in the 6‐well plate and treated firstly. After incubating with 4% paraformaldehyde for another 30 min, the cells were fixed, and incubated with 0.3% Triton X‐100 for approximately 5 min, then blocked for 20 min at 25°C. Anti‐CRT antibody was used to stained the cells for 30 min. Then, the cells were stained by secondary antibody and DAPI. The difference of anti‐HMGB1 from the above method of anti‐CRT stained was that cells were incubated by 0.3% Triton X‐100 at 25°C for 30 min. All the images were acquired by CLSM. Besides, when the cells were incubated with various treatments, they were incubated for another 4 h to Western blot analysis of HMGB1 expression.

### 2.12 Photoacoustic imaging

Photoacoustic performance of BSO/OVA@Fe‐GA was investigated by a multi‐modal imaging platform with a wavelength range from 680 to 970 nm. After acquiring the spectra of BSO/OVA@Fe‐GA, laser at 690 nm was chosen for the following PA imaging. The signal of water and BSO/OVA@Fe‐GA was examined at 690 nm.

### 2.13 In vivo CT26 orthotopic and bilateral tumor model

All animal procedures abided by the criterion of the Institutional Animal Care and Use Committee (IACUC) of the Animal Experiment Center of Guangzhou Medical University (Guangzhou, China) and were performed following institutional approval (Protocol GY2021‐048). Mice were housed under 12/12‐h light/dark cycle, keeping a constant temperature (21 ± 1°C) and relative humidity (40%–60%) environment, with free access to standard food and water. The experiment began after two weeks of quarantine.

4‐week‐old female BALB/c mice were applied for the experiment. First, one million CT26 cells were inoculated subcutaneously on the right side of each mouse, following the subcutaneous inoculation of five hundred thousand CT26 cells on the left side after a week. Once the tumor volume reached ≈100 mm^3^, the mice were randomly divided into 7 groups (PBS, OVA, BSO, BSO/Fe‐GA, OVA@Fe‐GA, BSO/OVA@Fe‐GA, and BSO/OVA@Fe‐GA + NIR). Then the mice were intravenously injected with 100 µL of various formulations. Laser irradiation at 1 W cm^−2^ was introduced to BSO/OVA@Fe‐GA + NIR 8 h post injection. Tumor volumes were recorded at 2 days’ interval.

### 2.14 In vivo antitumor immunity

The immune responses were measured using the single‐cell suspension prepared by the tumors and spleens after treatments. The cell suspension of tumors was stained with CD80/CD86 and investigated by FCM for DC cells activation analysis, and the cell suspension of tumors and spleens was stained with CD3^+^/CD4 ^+^ or CD3^+^/CD8 ^+^ for T cells activation analysis. Cells in tumor tissues were stained with CD4, CD25 and FoxP3 and investigated by FCM to detect Tregs amount. In addition, the cytokines levels in supernatants (TNF‐α, IFN‐γ, and IL‐6) were detected through ELISA kit assay.

## 3 RESULTS AND DISCUSSION

### 3.1 Characterization of photothermal nanoplatform

**FIGURE 1:**
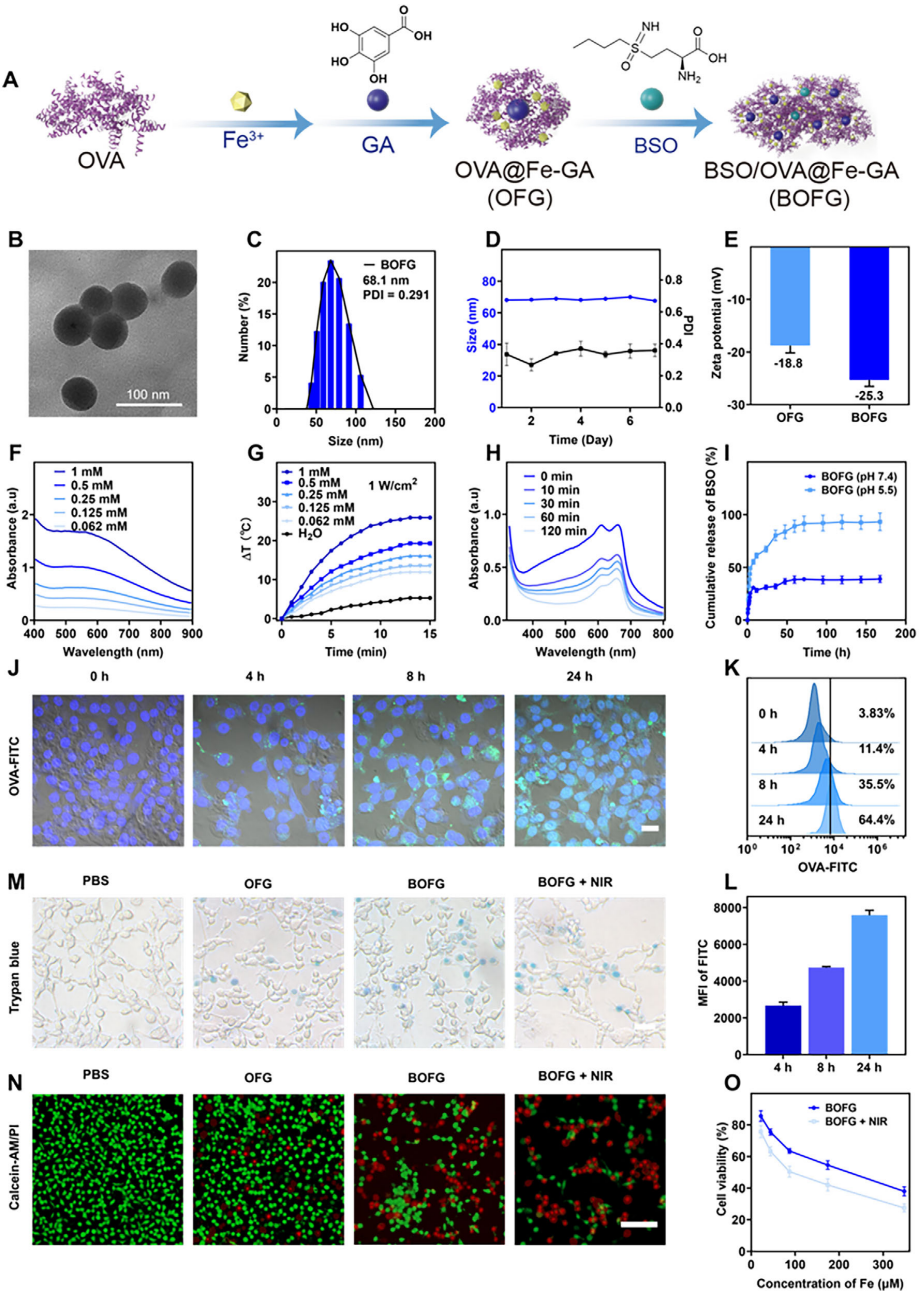
Preparation and characterizations of BOFG. (A) Synthesis process of BOFG. (B) TEM image of BOFG. (C) DLS‐measured size distribution of BOFG. (D) The long‐term stability of BOFG in aqueous medium. (E) Zeta potential data for OFG and BOFG. The data are shown as mean ± SD (*n* = 3). (F) UV–Vis spectra of BOFG at different concentrations. (G) Photothermal curves of BOFG with 808 nm laser irradiation at different concentrations. (H) Time‐dependent UV–Vis spectra of MB degradation triggered by Fenton reaction. (I) Drug release of BSO under different conditions. (J) Confocal laser scanning microscopy (CLSM) images, (K) flow cytometry results, and (L) mean fluorescence of CT26 cells after incubation with FITC‐labeled OVA (BOFG) for different periods. Scale bar = 20 µm. (M) Trypan blue exclusion assay of CT26 cells after 4 h of co‐incubation with PBS, OFG, BOFG, and BOFG + NIR (808 nm, 1 W cm^−2^, 5 min). Scale bar = 50 µm. (N) Calcein‐AM/PI live/dead cell staining analysis of CT26 cells after different treatments. Scale bar = 100 µm. (O) Cytotoxicity of BOFG with/without laser irradiation (808 nm, 1 W cm^−2^, 5 min) at different concentrations in CT26 cells for 24 h.

The model antigen OVA was applied here as the template for Fe^3+^‐GA MPNs adsorption to obtain OVA@Fe‐GA (OFG), then BSO as the irreversible inhibitor of GSH synthesis was loaded in via continuously stirring overnight to construct BOFG (Figure 1A). Transmission electron microscopy (TEM) images of BOFG displayed a uniform spherical shape with 50 nm in dehydrated diameter (Figure 1B). Meanwhile, the hydrodynamic diameter was 21 nm for OFG and 68 nm for BOFG, and their polydispersity indices are 0.365 and 0.291, and both of them displayed negative zeta potential by DLS analysis (Figure 1C,E; Figure S1, Supporting Information). The appropriate hydrodynamic diameters and surface charges enabled them to passively accumulate at tumor sites utilizing the enhanced permeability and retention (EPR) effect of solid tumors. The high hydrodynamic stability of BOFG was confirmed by tracking for 7 days in water (Figure 1D). The constitution of BOFG was confirmed by Inductively Coupled Plasma Optical Emission Spectrometer (ICP‐OES) and High‐Performance Liquid Chromatography (HPLC), calculated that Fe content is 4.38 mmol g^−1^ and BSO content is 300 mg g^−1^.

The detectable absorption of OFG and BOFG at the near‐infrared (NIR) wavelength in the UV–Vis spectra indicated their photothermal conversion effect (Figure 1F; Figure S2, Supporting Information). The temperature elevation of BOFG was monitored by infrared camera with 808 nm laser irradiation for different time. The temperature elevation is positively related to the concentration of BOFG, and the temperature of BOFG at Fe^3+^ concentration of 1 mm increased to 25.9°C under 1 W cm^−2^ of irradiation for 15 min (Figure 1G). The temperature variation was tracked by irradiation ON/OFF cycles to evaluate the photostability. The profile showed no changes during seven cycles, indicating the superior photostability of BOFG (Figure S3A, Supporting Information). The photothermal conversion efficiency (*η*) was calculated using the temperature recorded from one ON/OFF irradiation cycle every 10 s and the system time constant of 253.1 s, resulting that *η* of BOFG was 39.19% (Figure S3B, Supporting Information).^[^ ^15^ ^]^ All of them indicated the robust photothermal therapeutic potential of BOFG both in vitro and in vivo.

Fe ions release from BOFG in Acetate Buffer (ABS, pH = 5.5) via 1,10‐phenanthroline coloration reaction.^[^ ^16^ ^]^ Fe ions play a central role in catalyzing the intracellular Fenton reaction,^[^ ^17^ ^]^ thus the generated Fe ions in aqueous solution from BOFG were trapped by 1,10‐phenanthroline for quantitative analysis (Figure S4, Supporting Information). Then the Fenton reaction in aqueous solution was determined by the hydroxyl radical (·OH)‐induced methylene blue (MB) oxidation.^[^ ^18^ ^]^ The dark blue MB solution incubated with BOFG displayed a rapid fading during the first 10 min and became light blue at 2 h coincubation, indicating the effective generation of reactive oxygen species at the lysosome‐mimicking condition (ABS, pH = 5.5) (Figure 1H). Subsequently, the BSO release from BOFG was investigated by HPLC, and the cumulative release of BSO could reach 67.36% at 24 h (Figure 1I). With effective ROS generation from Fe^3+^‐GA MPNs‐mediated Fenton reaction and constant BSO release from the nanoplatform, BOFG showed great potential in intracellular GSH depletion.

### 3.2 Cellular uptake and cytotoxicity

CT26, an *N*‐nitroso‐*N*‐methylurethane‐induced undifferentiated colon carcinoma murine cell line, was utilized for in vitro investigation. Time‐dependent cellular uptake levels of BOFG were evaluated in CT26 cells by CLSM and flow cytometry analysis. In the CLSM images, the green fluorescence of FITC‐labeled OVA was weak at 4 h, displayed bright spot distribution in cytoplasm at 8 h, and became diffuse intracellular distribution at 24 h (Figure 1J; Figure S5, Supporting Information). This result indicated that effective uptake of BOFG took over 4 h, and internalized BOFG was relatively stable at 8 h. Meanwhile, flow cytometry analysis demonstrated that the FITC‐positive rates at different time points were 11.4%, 35.5% and 64.4%, respectively (Figure 1K; Figure S6, Supporting Information). And the mean fluorescent intensity was gradually promoted, matched well with the results of CLSM (Figure 1L). Based on these results, 8 h post‐incubation was selected as the time‐point of NIR irradiation in consideration of the fine uptake rate and structural stability.

The status of CT26 cells treated with OFG, BOFG and BOFG + NIR irradiation was observed by Trypan blue staining assays and Calcein‐AM/PI staining assays. As a cell‐impermeable dye, trypan blue could stain the cells with damaged cytomembrane. The optical images indicated that several OFG‐treated CT26 cells displayed blue nuclei, a few of BOFG‐treated CT26 cells lost membrane integrity, and a majority of cells in the BOFG + NIR irradiation group were stained (Figure 1M). It should be noted that 8 h incubation of OFG and BOFG without irradiation induced membrane breakage, which is unusual for typical photothermal nanoplatforms.^[^ ^19^ ^]^ Meanwhile, Calcein‐AM/PI staining confirmed the gradual death process of CT26 cells with damaged membranes, displaying the red fluorescence of PI in nuclei of dead cells. Without NIR irradiation, both OFG and BOFG could disturb the cell membrane of CT26 cells, and BSO supplement enhanced the destruction. NIR irradiation further promoted the membrane destruction of BOFG, and a majority of cells displayed stained nuclei by cell‐impermeable dyes (Figure 1N).

Subsequently, MTT assay was used to investigate the concentration‐dependent cytotoxicity of BOFG without and with NIR irradiation. At 8 h post‐incubation, CT26 cells were irradiated at 1 W cm^−2^ for 5 min and incubated for another 16 h before MTT addition. Over 70% of irradiated cells were dead at a high Fe^3+^ concentration of 348 µm, indicating the superior antitumor effect. Without NIR irradiation, BOFG can also inhibit tumor cell growth with a concentration‐dependent manner, the cell viability was below 50% at Fe^3+^ concentration of 348 µm (Figure 1O).

**FIGURE 2:**
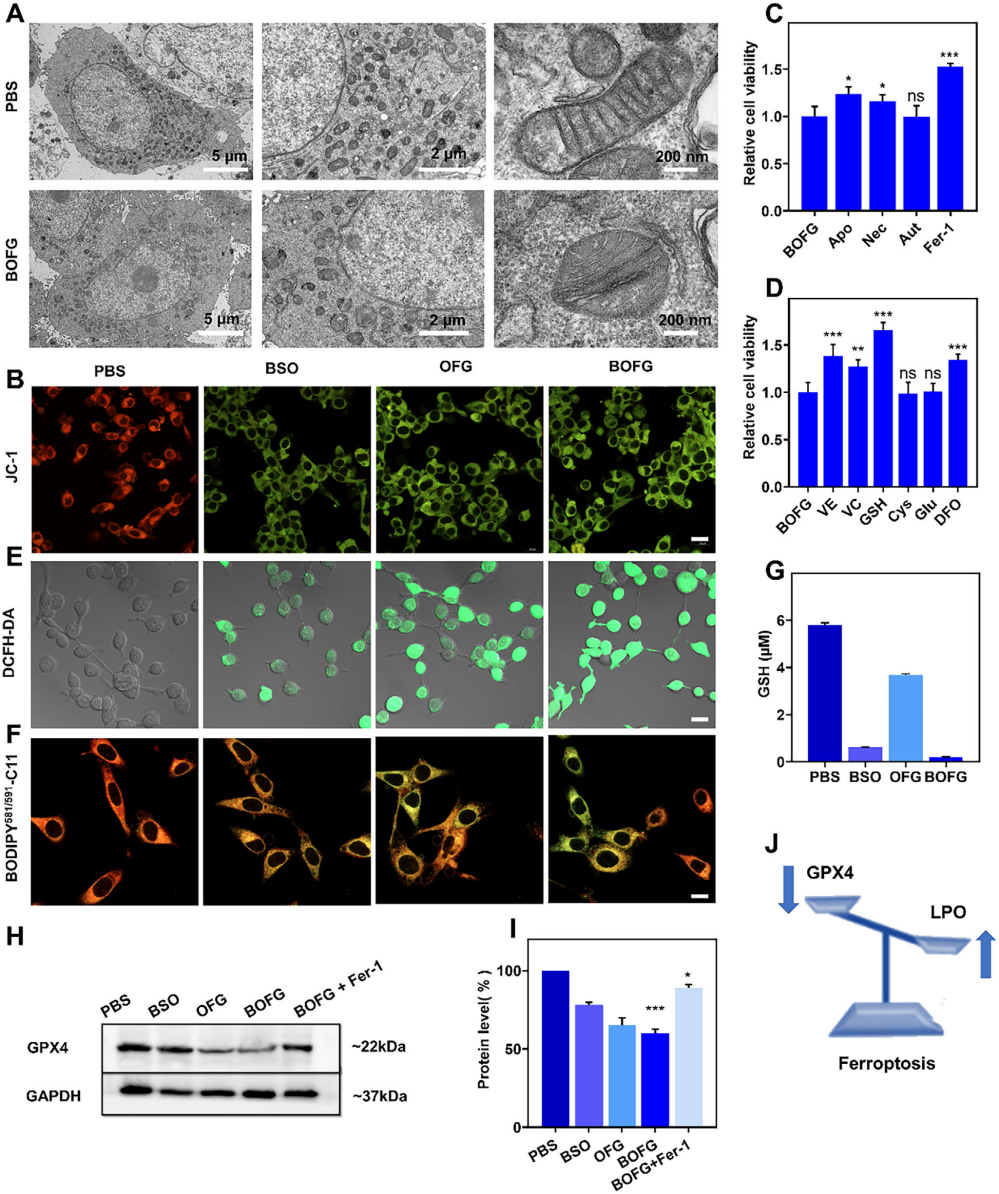
Cellular uptake and cytotoxicity. (A) Bio‐TEM images of PBS and BOFG‐treated CT26 cells were displayed at 8 h post‐incubation, and the figures with different magnifications were shown with the scale bars of 5 µm, 2 µm, and 200 nm. (B) CLSM images of JC‐1 assay detecting mitochondrial membrane potential of CT26 cells after various treatments. Scale bar = 20 µm. (C) Viability of CT26 cells treated with BOFG ± Ac‐DEVD‐CHO (Apo, 50 µm), Necrostatin‐1 (Nec, 490 nm), 3‐methyladenine (Aut, 60 µm), and ferrostatin‐1 (Fer‐1, 100 nm). (D) Viability of CT26 cells treated with BOFG ± vitamin E (VE, 20 µm), sodium ascorbate (VC, 20 µm), glutathione (GSH, 1 mm), cystine (Cys, 1 mm), glutamic (Glu, 1 mm), and deferoxamine (DFO, 100 µm). (E) CLSM images of DCFH‐DA assay detecting intracellular ROS level of CT26 cells after various treatments. Scale bar = 20 µm. (F) CLSM images of BODIPY581/591‐C11 assay detecting lipid peroxides of CT26 cells after various treatments. Scale bar = 10 µm. (G) Intracellular GSH levels of CT26 cells treated with PBS, BSO, OFG, and BOFG. The GSH concentration was detected using Elman's reagents. (H,I) Expression of GPX4 in CT26 cells with different treatments for 24 h. (J) Schematic illustration of BOFG induced ferroptosis. n.s. represented no significance, *p < 0.05, **p < 0.01, ***p < 0.001.

Therefore, the death process of these unirradiated cells in BOFG group was further investigated in CT26 cells. Bio‐TEM images of BOFG‐treated CT26 cells displayed shrunken mitochondria with dense membrane, mitochondria cristae missing, normal cell nucleus without chromatin condensation at 8 h post‐incubation, exhibiting the typical morphological changes of ferroptotic cell death (Figure 2A). The mitochondria status was further observed by JC‐1 staining. Obvious mitochondrial damage was confirmed by the generation of JC‐1 monomers with green fluorescence in cells of OFG and BOFG groups (Figure 2B).

To better understand the death mechanism of BOFG‐treated CT26 cells, inhibitors of various cell death pathways and several modulators of ferroptosis were utilized to regulate cell viability in MTT assays. 3‐methyladenine (Aut, an autophagy inhibitor) could hardly rescue CT26 cells from death, while Ac‐DEVD‐CHO (Apo, an apoptosis inhibitor), necrostatin‐1 (Nec, a necroptosis inhibitor), and ferrostatin‐1 (Fer, inhibitor of ferroptosis) obviously promoted their viability (Figure 2C; Figure S7, Supporting Information). With the addition of the iron chelating agent deferoxamine (DFO) to remove the intracellular Fe ions, the viability was also elevated. Meanwhile, both Vitamin E (VE, cytoplasmic LPO scavenger) and Vitamin C (cytoplasmic ROS scavenger) as the typical reductants can alleviate the toxicity of BOFG (Figure 2D). These indicated that the iron supply and oxidative stress were important for BOFG to function. And it should be noted that GSH addition could rescue the cells, but the cystine (Cys) or glutamate (Glu) addition failed to work (Figure 2D; Figure S7, Supporting Information). That is because BSO, as the inhibitor of c‐glutamylcysteine synthetase (c‐GCS), is independent on the upstream Cys and Glu supply for intracellular GSH depletion.^[^ ^20^ ^]^ Moreover, the intracellular GSH level was measured by GSH and GSSG detection kits. The application of BSO had dramatically downregulated the intracellular GSH in the groups of BSO and BOFG, while the OFG slightly reduced the GSH level (Figure 2G). These results further demonstrated the GSH synthesis inhibition mediated by BSO, implying the increase of intracellular oxidative stress of CT26 cells.

The intracellular ROS and lipid peroxides (LPO) levels are crucial indexes for ferroptotic cell death. DCFH‐DA probe was applied to evaluate the intracellular ROS levels, and the CLSM images displayed obvious green fluorescence in cells treated with BSO and OFG, and bright green fluorescence in cells treated with BOFG (Figure 2E). And the relative flow cytometry analysis demonstrated the DCF‐positive rates of BSO, OFG and BOFG groups were 7.79%, 12.7%, and 36.2%, respectively (Figure S8A,B, Supporting Information). And the MFI of BOFG group was the highest among the three groups (Figure S8C, Supporting Information). The lipid peroxidation‐sensitive probe BODIPY^581/591^‐C11, featuring with significant maximum fluorescence emission peak shift, of which from 590 nm to 510 nm after oxidation by LPO, was applied to evaluate the LPO levels. CLSM images displayed the robust yellow fluorescence merged from low ratio of the reduction state with red fluorescence and high ratio of the oxidation state with green fluorescence in the cells of BSO, OFG, and BOFG groups, indicating the intracellular LPO accumulation after the treatments (Figure 2F). Then, the expression of GPX4 in CT26 cells was further investigated after different treatments. Western blotting assays indicated that the treatment of BSO, OFG, and BOFG inhibit the GPX4 expression, while Fer‐1 addition reversed the tendency of BOFG treatment (Figure 2H,I). Overall, BOFG induced CT26 cell death via multiple pathways, including ferroptosis as the dominant pathway (Figure 2J).

### 3.3 In vitro immune activation

**FIGURE 3:**
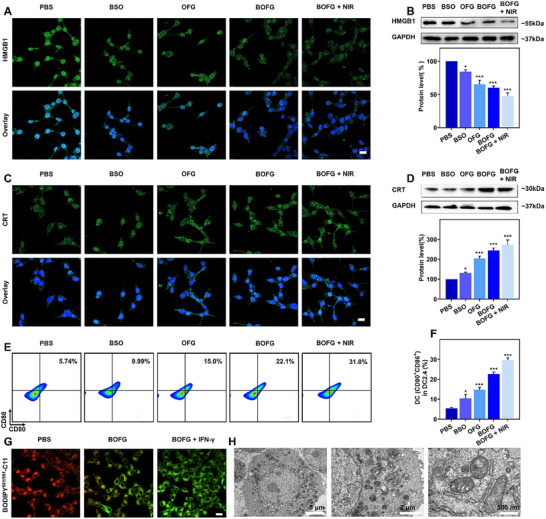
In vitro immune activation. (A) HMGB1 release from CT26 cells treated with different treatments for 8 h. Scale bar = 20 µm. (B) Measurement of HMGB1 expression by western blot in CT26 cells cultured with different treatments for 8 h. (C) Surface exposed CRT on CT26 cells treated with different treatments for 8 h. Scale bar = 20 µm. (D) Measurement of CRT expression by western blot in CT26 cells cultured with different treatments for 8 h. (E) Flow cytometry analysis of the expression of CD80 and CD86 on the surface of DCs after different treatments. (F) Quantitative analysis of the DC maturation level. (G) CLSM images of BODIPY581/591‐C11 assay detecting lipid peroxides of CT26 cells after various treatments. Scale bar = 20 µm. (H) Bio‐TEM image of BOFG + IFN‐γ treated CT26 cells at different magnifications with the scale bars of 5 µm, 2 µm, and 500 nm. n.s. represented no significance, *p < 0.05, **p < 0.01, ***p < 0.001.

Both photothermal effect‐induced apoptosis and ferroptosis‐mediated cell death can elicit ICD of the treated CT26 cells, which would release DAMP signals involving HMGB1 emission and CRT translocation onto the surface. Therefore, HMGB1 and CRT in the cells were investigated via immunofluorescent staining and western blotting assays. HMGB1 as a highly conserved nuclear protein would be released to extracellular matrix upon cell damage, thus promoting the maturation of local DCs.^[^ ^21^ ^]^ In CLSM images, the intranuclear green fluorescence of HMGB1 stained by FITC‐anti HMGB1 was gradually decayed in the groups of PBS, BSO, OFG, BOFG and BOFG + NIR in sequence (Figure 3A). And western blotting results confirmed the similar expression tendency of HMGB1 among the five groups (Figure 3B). Semi‐quantitative analysis indicated that over 50% of HMGB1 was emitted from the BOFG‐treated cells under NIR irradiation (Figure 3B). Meanwhile, CRT that localized at the endoplasmic reticulum would translocate to the outer cell surface and release an “eat me” signal to accelerate the phagocytosis by DCs.^[^ ^22^ ^]^ In CLSM images, the green fluorescence of CRT stained by FITC‐anti CRT was more robust in the cells treated with BSO, OFG, BOFG or BOFG + NIR than the cells in PBS group (Figure 3C). The corresponding western blotting results demonstrated that BOFG treatment without or with NIR irradiation could double the expression of CRT, implying the occurrence of ICD that would recruit DCs to present these tumor‐associated antigens (TAAs) (Figure 3D). The high expression of CD80 and CD86 as hallmarks of DC maturation portended the successful immune stimulation. The treated CT26 cells of different groups were added in the upper chamber of transwells and incubated with murine DC2.4 cells in the bottom chamber. Mature DCs (CD11c^+^CD80^+^CD86^+^) were collected for flow cytometry analysis (Figure 3E). The population of mature DCs in BSO was ≈1.74‐fold higher than that in PBS group (5.74%), suggesting BSO‐induced GSH‐depletion increased DC maturation rate. While OFG and BOFG enhanced DC maturation to ≈2.61‐fold and ≈3.85‐fold higher than that from PBS group, ascribing to the inherent OVA antigenicity, Fe^3+^‐mediated Fenton reaction, and BSO‐inhibited GSH synthesis (Figure 3F). Dramatic DC maturation was promoted to the highest level in BOFG + NIR group, indicating the highest level of DAMPs release of CT26 cells with this treatment. As the antigen presentation is a key index of DC maturation, the expression of H‐2Kb‐SIINFEKL complexes was utilized to evaluate the presentation of the model antigen OVA, reflecting the TAAs and tumor specific antigens (TSAs) presentation tendency of the treated CT26 cells. Results showed that BOFG + NIR increased the proportion of SIINFEKL antibody‐positive cells by 1.92‐ and 1.52‐fold compared to OFG and BOFG, respectively (Figure S9, Supporting Information). The much higher MFI also confirmed the superior antigen presentation of DCs incubated with CT26 cells in BOFG + NIR group, indicating the enhanced T cell activation (Figure S10, Supporting Information).

DC maturation would initiate the CD8^+^ T cell‐involved cancer‐immunity circle, and the activated CD8^+^ T cells would release interferon gamma (IFN‐γ), which was capable of downregulating the expression of solute carrier 7A11 (SLC7A11) and solute carrier 3A2 (SLC3A2), two subunits of the glutamate‐cystine antiporter system x_c_^−^. This would impair the system x_c_^−^ to internalize cystine, reduce the GSH synthesis, and suppress the activity of glutathione peroxidase 4 (GPX4), thus facilitate lipid peroxidation and subsequent ferroptosis induction. On the basis of these knowledge, we evaluated the function of IFN‐γ in BOFG‐treated CT26 cells. CLSM images displayed robust green fluorescence of oxidative BODIPY 581/591‐C11 in the cells treated with BOFG and IFN‐γ, indicating the intracellular LPO accumulation with higher levels than that of BOFG‐treated cells (Figure 3G). Typical morphology changes of ferroptosis were found in bio‐TEM images of cells in BOFG + IFN‐γ group, displaying shrunken mitochondria with dense membrane, mitochondria cristae missing (Figure 3H). Therefore, the active CD8^+^ T cells in antitumor immunity would promote BOFG‐induced ferroptotic cell death via the release of IFN‐γ.

### 3.4 In vivo antitumor therapy

**FIGURE 4:**
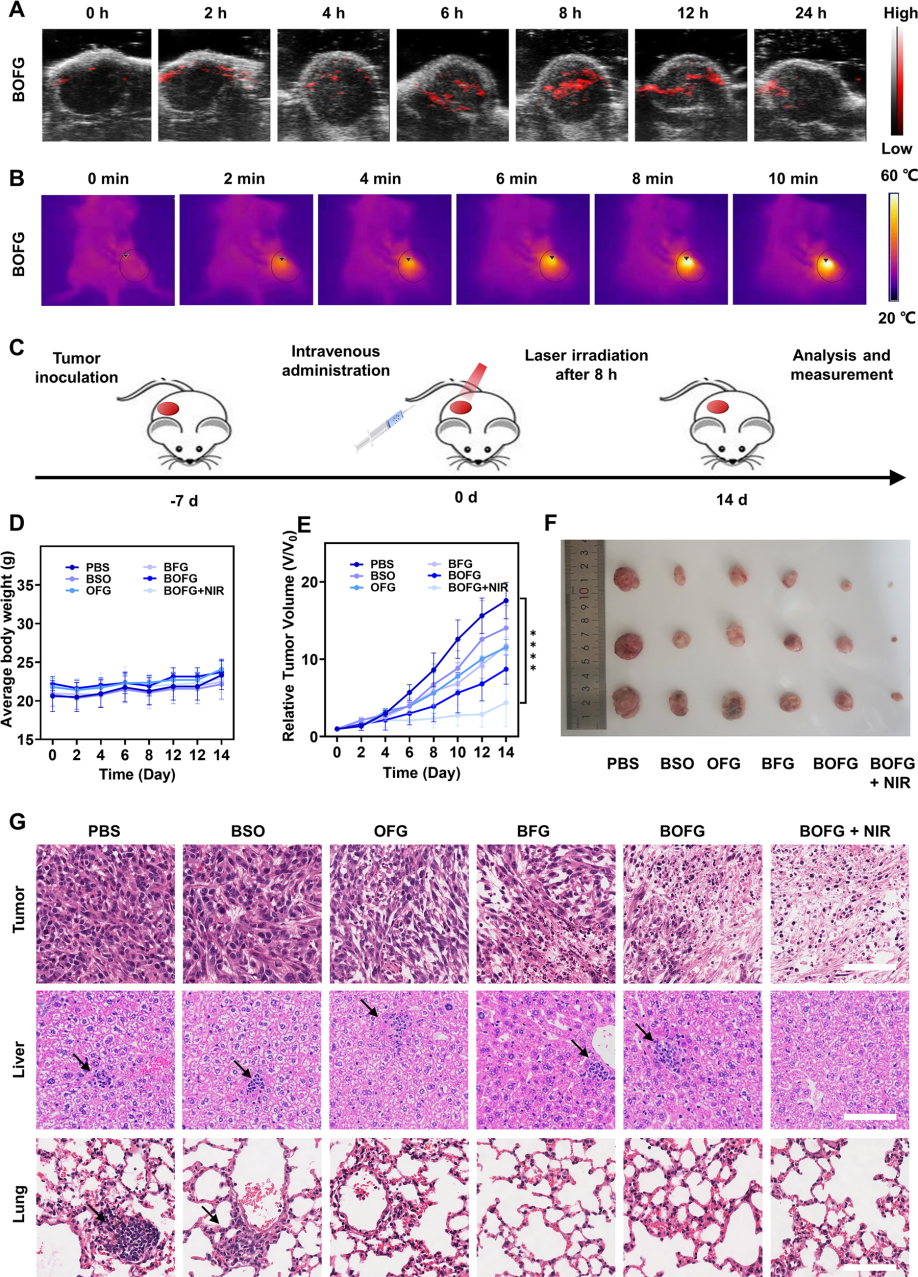
In vivo antitumor therapy. (A) PA images at tumor site post‐injection. (B) Temperature variation of mice at tumor site with laser irradiation at a density of 1 W cm^−2^ for 10 min, 8‐h post‐injection of BOFG. (C) Time schedule of BOFG for tumor inhibition. (D) Changes in body weight of mice with different treatment. Data are shown as the mean values ± SD (*n* = 3). (E) The tumor volume changes of the mice after different treatment. Data are shown as the mean values ± SD (*n* = 3). (F) Images of CT26 tumors from different groups. (G) H&E staining images of tumor, liver, and lung after various treatments. Scale bar = 100 µm. n.s. represented no significance, *p < 0.05, **p < 0.01, ***p < 0.001, ****p < 0.0001.

In vivo evaluation was start with photoacoustic (PA) imaging.^[^ ^23^ ^]^ Owing to the high absorbance in NIR region, BOFG displayed fine PA contrast in solution with a concentration‐dependent manner (Figure S11, Supporting Information). Then, ectopic CT26 tumor‐burden BABL/C mice were intravenously injected with 100 µL of BOFG (at the Fe concentration of 3.48 mm) and monitored the PA signal of tumor site at the pre‐determined time points. The highest PA signal was observed at 8 h post‐injection, implying the high potency of NIR irradiation‐mediated photothermal conversion (Figure 4A). Therefore, photothermal imaging was applied to investigate the temperature elevation process at 8 h post‐injection of BOFG. With 1 W cm^−2^ of NIR irradiation for 10 min, the tumor temperature showed negligible overall elevation (≈5°C) in mice of PBS group, while the tumor of mice treated with BOFG kept over 50°C since the third minute, indicating the superior photothermal therapeutic outcome (Figure 4B; Figure S12, Figure S13, Supporting Information). Subsequently, in vivo primary tumor inhibition was employed to evaluate the therapeutic outcome of BOFG without or with NIR irradiation.

Female BALB/c mice bearing ≈100 mm^3^ of ectopic CT26 tumor on the lateral side of the right hind leg were randomly divided into six groups. The therapeutic agents were intravenously injected into tail vein of mice at day 0, and NIR irradiation was applied on the tumor site of mice in BOFG + NIR group at 8 h post‐injection (Figure 4C). On the day 14, the data tracking ended due to the biggest tumor of PBS group became too burdensome. The mice body weight change of each group showed insignificant variation during the half‐month treatment course (Figure 4D). During this process, BOFG with NIR irradiation displayed obvious tumor inhibition and the average tumor volume was below 300 mm^3^, while BOFG without NIR irradiation showed suboptimal tumor inhibition and the average tumor volume increased to ≈800 mm^3^. BFG that obtained from coprecipitation of BSO, Fe^3+^ and GA displayed similar antitumor effect of OFG, and the tumor volume of both groups increased to 12 times of the initial tumor volume. Free BSO showed negligible tumor inhibition due to the rapid clearance of small molecules in blood (Figure 4E). All mice were euthanized, and tumors were excised, weighed and photographed (Figure 4F). The tumors and main organs were fixed for further analysis by hematoxylin and eosin (H&E) staining. Tumors of the PBS and BSO groups could be observed the closely packed cells while a fraction cells displayed nuclei missing in tumors of the OFG and BFG groups, while partial nuclei destruction was detected in tumors of the BOFG group, and remarkable shrunken or anuclear cells were observed in tumors of the BOFG + NIR group (Figure 4G). These results matched well with the therapeutic outcome tendency of each group. Pathological abnormalities were indetectable in H&E staining images of the heart, spleen, and kidney of the mice in the six groups (Figure S14, Supporting Information). However, some small tumor nodules were found in the liver and lung of the mice after the different treatments without NIR irradiation, which hinted the insufficient initiation of tumor immunity cycle via the single therapy and thus metastasis inhibition failure (Figure 4G).

### 3.5 In vivo anti‐metastasis immunotherapy

**FIGURE 5:**
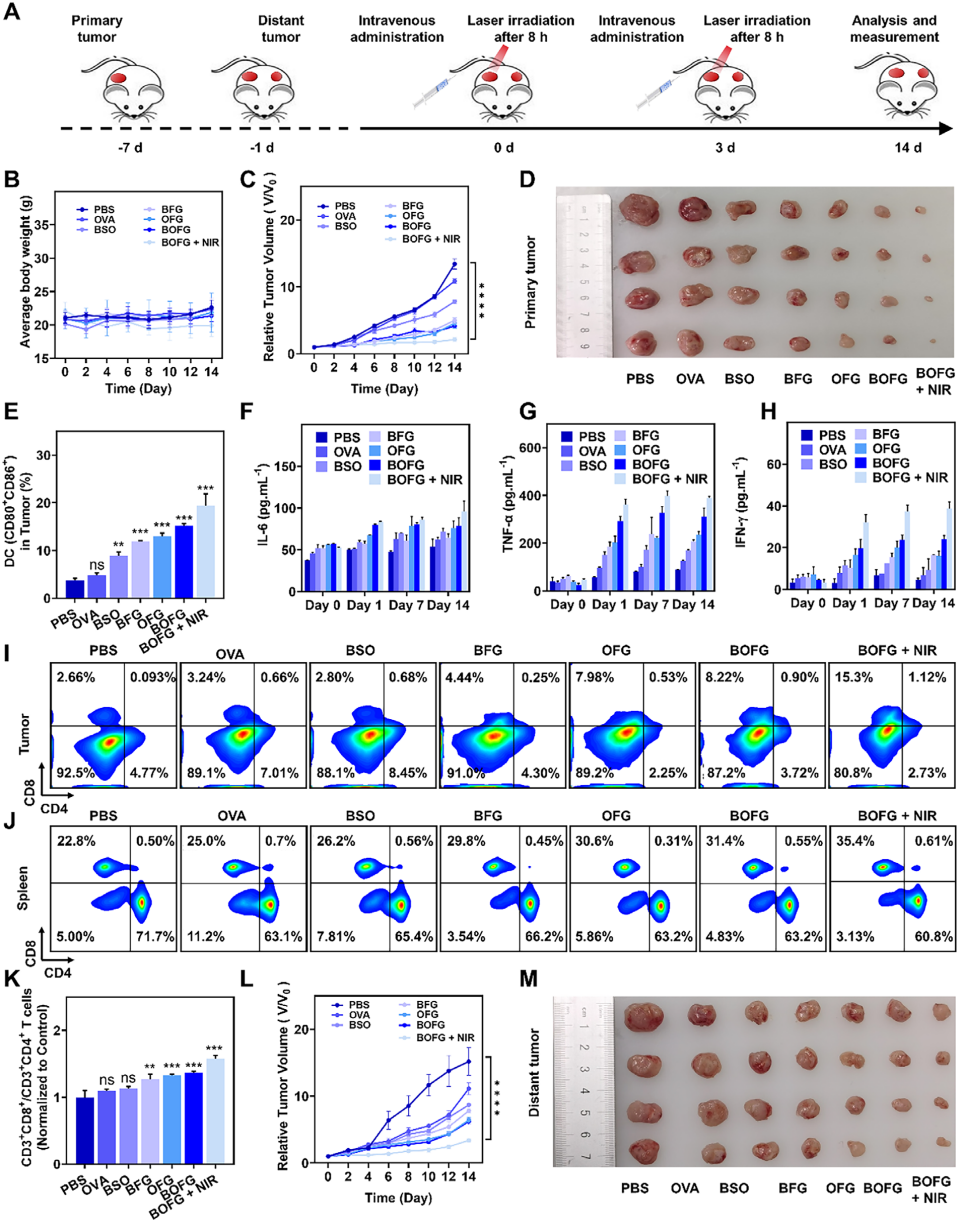
In vivo anti‐metastasis immunotherapy. (A) Schematic illustration the application of BOFG for the treatment of primary and distant tumor. (B) Changes in body weight of mice with different treatment. Data are shown as the mean values ± SD (*n* = 4). (C) The primary tumor volume changes of the mice after different treatment. Data are shown as the mean values ± SD (*n* = 4). (D) Images of primary tumor from different treatments. (E) The absolute percentages of CD80 and CD86 on the surface of DCs in tumor after different treatments. (F–H) Cytokine levels of IL‐6, TNF‐α, and IFN‐γ are shown from serum of mice collected at 0 (before), 1, 7, and 14 days after the different treatments. (I) A representative flow cytometry analysis displaying the absolute percentage of CD8^+^ T cells and CD4^+^ T cells in CD3^+^ tumor‐infiltrating T lymphocytes from mice with different treatments. (J) A representative flow cytometry analysis displaying the absolute percentage of CD8^+^ T cells and CD4^+^ T cells in CD3^+^ splenic cells from mice with different treatments. (K) The distant tumor volume changes of the mice after different treatment. Data are shown as the mean values ± SD (*n* = 4). (L) Distant tumor weights in mice given different treatments. Data are presented as the mean ± SD (*n* = 4). (M) Images of distant tumor from different treatments. n.s. represented no significance, *p < 0.05, **p < 0.01, ***p < 0.001, ****p < 0.0001.

Since CT26 cells are extremely mobile and adept at penetrating surrounding tissue to form metastatic tumor nodules at distant tissue, the practical antitumor therapy usually started long after small distant metastases formation. Based on the previous results, the in vivo anti‐metastatic effect of the nanoplatforms was further investigated. Female BALB/c mice were subcutaneously injected with 5 × 10^6^ of CT26 cells on the lateral side of the right hind leg to form the primary tumor and injected with 1 × 10^6^ of CT26 cells on the lateral side of the left hind leg after 6 days to mimic the distant metastasis. The mice were randomly divided into seven groups on the next day. Various recipes were intravenously injected into tail vein on the day 0 and day 3, and NIR irradiation on the tumor sites was implemented 8 h after the BOFG administration in the group of BOFG + NIR (Figure 5A). During the therapeutic period, no remarkable fluctuations of body weight profile were observed in the seven groups (Figure 5B). And the therapeutic outcome of the primary tumor showed similar tendency of the above single therapy, while the primary tumor growth profile of the new therapy with OVA‐treated mice almost coincided with that of PBS group from day 0 to day 12. Inspiringly, dual application of BFG, OFG, BOFG could effectively inhibited the tumor growth, and the average tumor volume increased to ≈5.01‐fold, ≈4.57‐fold, and ≈4.16‐fold of their initial tumor volume. Significant primary tumor inhibition was found in mice treated with BOFG and NIR irradiation, and the final tumor volume were all below 50 mm^3^ (Figure 5C). On the day 14, all mice were euthanized, and tumors on both legs were excised, weighed and photographed. The primary tumors were collected for a series of analyses (Figure 5D; Figure S15, Supporting Information).

For further therapeutic evaluation, the tumor paraffin sections were assayed with H&E staining, Ki‐67 antibody staining and terminal deoxynucleotidyl transferase mediated dUTP nick‐end labeling (TUNEL) staining for histological analysis, cell apoptotic analysis, and proliferative activity analysis, respectively. In the H&E images, densely packed cells were found in tumors of the PBS and OVA groups, minor anuclear cells were shown in tumors of BSO group, a fraction cells displayed nuclei missing in tumors of the OFG and BFG groups, while partial cell destruction was detected in tumors of the BOFG group, and abundant anuclear cells were observed in tumors of the BOFG + NIR group (Figure S16, Supporting Information). TUNEL staining results indicated that the treatments of BOFG without or with NIR irradiation had dramatically increased the cell apoptotic rate (Figure S17, Supporting Information). And Ki67 expression of the CT26 cells were downregulated by various treatments, and reduced to below one third in the groups of OFG, BOFG, and BOFG + NIR (Figure S18, Supporting Information).

For local immune activation analysis, the homogenized primary tumors were filtered to obtained single‐cell suspension for flow cytometry analysis. The population of mature DCs (CD11c^+^CD80^+^CD86^+^) in OVA group (4.30%) was at the same level of PBS group (3.54%), implying that the intravenous administration of single OVA antigen failed to alter the tumor immune microenvironment. While BSO and BFG enhanced DC maturation to ≈2.70‐fold and ≈3.31‐fold higher than that from PBS group, ascribing to ICD occurrence mediated by the BSO‐inhibited GSH synthesis and Fe^3+^‐mediated Fenton reaction. Dramatic DC maturation was promoted to 4.23‐fold and 5.48‐fold in BOFG group and BOFG + NIR group, indicating the high level of DAMPs release by primary CT26 tumors with these treatments (Figure 5E; Figure S19, Supporting Information). After DC maturation, the antitumor immunity would be elicited via varying intratumoral T cell populations. Since CD8^+^ T cells are the main T‐cell subset responsible for antitumor immune response, the increasing of CD8^+^ T cells infiltration indicated the potential of generating potent antitumor immune response. OFG and BOFG treatments showed much higher infiltration of CD8^+^ T cells (7.98% and 8.22%, respectively) in tumors treated with PBS (2.66%), OVA (3.24%), BSO (2.80%), and BFG (4.44%). And NIR irradiation significantly increased the infiltration of CD8^+^ T cells to 15.3% in tumors of BOFG + NIR group, which were also demonstrated by the most robust green fluorescence of CD8^+^ T cells in the tumor sections. While the CD4^+^ T cell infiltration displayed ambiguous tendency in tumors after different treatments, and the BOFG + NIR treatment showed low infiltration of CD4^+^ T cells (2.73%) (Figure 5I; Figure S20, Supporting Information). Therefore, a high population ratio of CD8^+^ T cells to CD4^+^ T cells was found in the group of BOFG + NIR, illustrating that CD8^+^ T cells played a dominant role during the treatment. In addition, regulatory T cells (Tregs), as an important type of immunosuppressive cell that expresses the nuclear transcription factor Forkhead Box P3 (FoxP3) biomarker, generally suppressed tumor immunity after infiltration.^[^ ^24^ ^]^ BOFG + NIR therapy was confirmed to decrease Tregs differentiation to 5.83% for enhanced antitumor immunity. Especially, the abundance of CD3^+^CD4^+^Foxps^+^ Tregs in tumors significantly decreased in BOFG group (12.8%) compared to the groups of OFG (14.4%), BSO (17.3%), OVA (24.2%), and PBS (33.1%) (Figures S21 and S22, Supporting Information).

For systemic immune activation analysis, the serum levels of pro‐inflammatory cytokines including interleukin‐6 (IL‐6), tumor necrosis factor α (TNF‐α), and interferon‐γ (IFN‐γ) were measured via ELISA and the homogenized spleens were filtered to obtained single‐cell suspension for flow cytometry analysis. Mice treated with BOFG + NIR showed the highest serum levels of IL‐6, TNF‐α, and IFN‐γ, which evidenced that this treatment induced effective cellular immune responses (Figure 5F–H). The population of T cells in the spleens varied among different treatments. BFG, OFG and BOFG treatments showed much higher ratio of CD8^+^ T cells (29.8%, 30.6%, and 31.4%, respectively) in splenic treated with PBS (22.8%), OVA (25%), and BSO (26.2%). And NIR irradiation further increased the ratio of CD8^+^ T cell subset to 35.4% in tumors of BOFG + NIR group, suggesting a stronger upregulation in splenic CD8^+^ T cells (Figure 5J). Compared with PBS group, a slight increase of the population ratio of CD8^+^ T cells to CD4^+^ T cells was found in the spleens of the other six groups (Figure 5K; Figure S23, Supporting Information).

Encouraged by the above high ratio of activated CD8^+^ T cells, the tumor sections were stained and observed for ferroptosis investigations. IFN‐γ released from CD8^+^ T cells could downregulate the expression of SLC7A11 and impair cysteine uptake by tumor cells, thereby inhibiting intracellular GSH synthesis and triggering lipid peroxidation accumulation in tumor cells, which consequently resulted in ferroptosis. The tumors with BOFG + NIR treatment have recruited the largest population of activated CD8^+^ T cells, displaying the lowest ratio of GPX4‐positive cells and the weakest fluorescence of SLC7A11 (Figures S24 and S25, Supporting Information). Both results indicated the occurrence of ferroptosis within the tumor. The high level of IFN‐γ expression was also found in the tumors of this group (Figure S26, Supporting Information). Effective distant tumor inhibition was also confirmed by the data of relative tumor volume, and tumor images (Figure 5L,M). All of these demonstrated the superior anti‐metastasis properties of BOFG + NIR treatment. Pathological abnormalities were indetectable in H&E staining images of the heart, liver, spleen, lung, and kidney of the mice in the seven groups (Figure S27, Supporting Information). Furthermore, blood biochemistry and blood routine analysis of the mice were performed after treatments to evaluate the biosafety of BOFG. The levels of aspartate transaminase (AST), alanine aminotransferase (ALT), creatinine (CRE), UREA, and total bilirubin (TBIL) maintained normal conditions after various treatments, suggesting the negligible hepatic and renal function damage (Figure S28, Supporting Information). The hematological parameters were in the normal ranges with an acceptable blood compatibility (Figure S29, Supporting Information).

Therefore, we summarized that the photothermal effect under NIR irradiation, the Fenton reaction catalyzed by Fe^3+^‐GA MPNs and the BSO‐blocked GSH synthesis collectively facilitate BOFG to induce ICD of CRC. Then, the released TAAs would promote DCs antigen‐presentation and maturation, and thus boost T cells proliferation and infiltration. Subsequently CD8^+^ T cells release IFN‐γ to inhibit neighboring tumor cells uptaking cystine for GSH synthesis, which further enhance the suppression of GPX4 and sensitize the cell to immunogenic ferroptosis inducing by Fe^3+^ and BSO. Finally, the tumor cells at intertumoral and intratumoral levels would be involved in the ferroptosis‐dominated cancer‐immunity circle for complete CRC ablation.

## 4 CONCLUSION

In this work, a photothermal nanoplatform with BOFG to initiate the closed loop of ferroptosis induction and immunotherapy under controllable NIR irradiation was constructed. By harnessing the photothermal conversion effect of Fe^3+^‐GA MPNs, BOFG is capable of initiating PTT under highly controllable NIR irradiation. Unlike previously reported photothermal nanoplatforms, model antigen ovalbumin possessing intrinsic immunogenicity was exploited as the template of MPN formation, obtaining small‐sized OFG. The c‐GCS inhibitor BSO was added to the previous recipe to obtain BOFG with ≈68 nm in diameter, thus satisfying the size needs of EPR effect and intratumoral accumulation. Interestingly, BOFG induced CT26 cell death via multiple pathways, including ferroptosis as the dominant pathway. Moreover, in vitro immune activation assays demonstrated the ICD occurrence mediated by BOFG without or with NIR irradiation. Meanwhile, both in vivo antitumor and anti‐metastasis assays demonstrated the effective tumor suppression of BOFG after NIR irradiation.

Although subcutaneous CT26 solid tumor could mimic the growth of CRC, the orthotopic implanted or induced primary tumor models would facilitate the understanding of immune activation in the practical therapy of CRC. The strategies of in combination with the targeting agents that can promote their tumor accumulation and incorporating adjuvants agents or checkpoint blockade inhibitors to facilitate the initiation of the therapeutic cascade loops would be the feasible and appealing improvements for the current nanoplatform. Looking forward, our strategy employing a photothermal nanoplatform to rapidly stimulate initiation of ICD and suppress the oxidation defense system represents a promising new method to efficiently enhance the “cascade loop” of ferroptosis induction and immunotherapy for treatment of drug resistant tumors.

## CONFLICT OF INTEREST STATEMENT

The authors declare no conflicts of interest.

## Supporting information

Supporting Information

## Data Availability

The data that supports the findings of this study are available in the supplementary material of this article.
