# Supplementary material for: Cascade loop of ferroptosis induction and immunotherapy based on metal‐phenolic networks for combined therapy of colorectal cancer
Source: Exploration (Beijing). 2024 May 15;5(1):20230117. doi: 10.1002/EXP.20230117 (PMC11875444; doi:10.1002/EXP.20230117)
Supplement: Supplementary file 1 — Supporting Information [file EXP2-5-20230117-s001.pdf]

# Supporting Information

## Cascade loop of ferroptosis induction and immunotherapy based on metal-phenolic networks for combined therapy of colorectal cancer

Yuwei Li[1,†], Yuxi Duan[1,†], Yunyi Li[2,†], Yuan Gu[1], Lu Zhou[1], Zhongting Xiao[1], Xinying Yu[1], Yanjun Cai[1], Erzhuo Cheng[1], Qianqian Liu[1], Yong Jiang[1], Quan Yang[1], Feng Zhang[1,*], Qi Lei[3,*] and Bin Yang[1,*]

[1]School of Biomedical Engineering, The Fourth Affiliated Hospital of Guangzhou Medical University, Guangzhou Medical University, Guangzhou, People's Republic of China
[2]Department of Nephrology, First Affiliated Hospital of Jinan University, Guangzhou, People's Republic of China
[3]The Second Affiliated Hospital, Provincial Key Laboratory of Allergy & Clinical Immunology, Guangzhou Medical University, Guangzhou, People's Republic of China

†These authors contribute equally to this article.

**Corresponding Authors**
E-mail: bin.yang@gzhmu.edu.cn (B. Yang),
leiqi@gzhmu.edu.cn (Q. Lei),
fzhang@usst.edu.cn (F. Zhang).

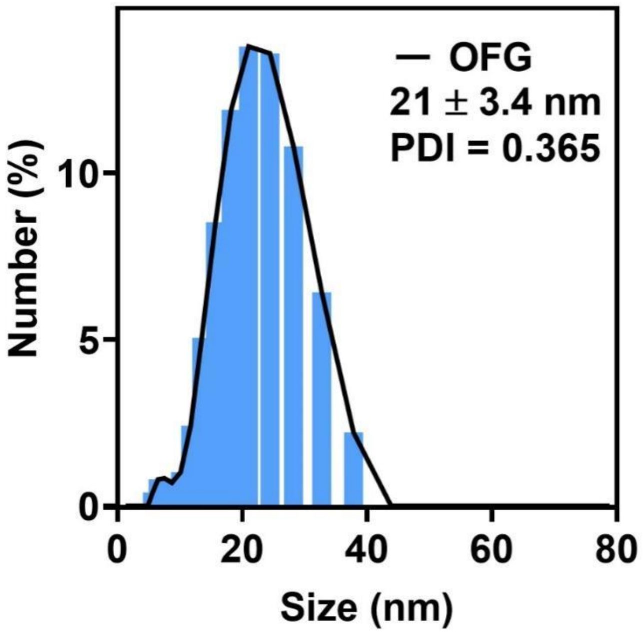


**Figure S1.** Hydrodynamic size of OFG measured by DLS.

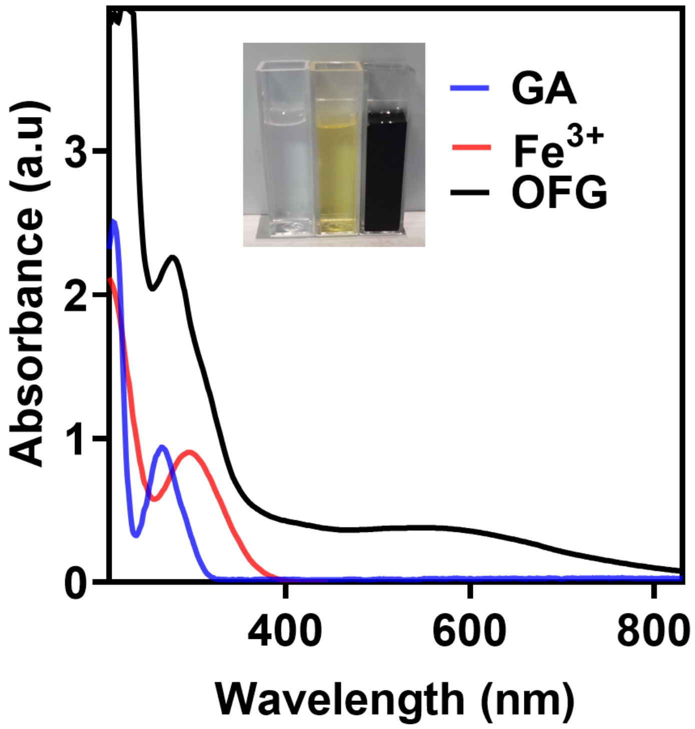


**Figure S2.** UV-Vis spectra of GA, $Fe^{3+}$ and OFG.

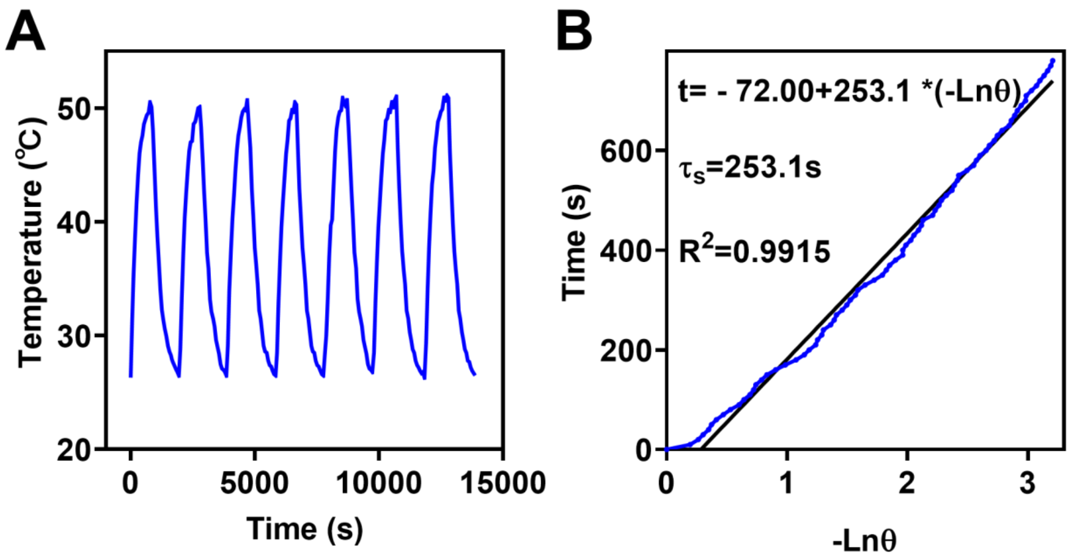


**Figure S3.** A) Calculation of the photothermal conversion efficiency at 808 nm. B) Continuous irradiation-cooling cycle profiles of BOFG.

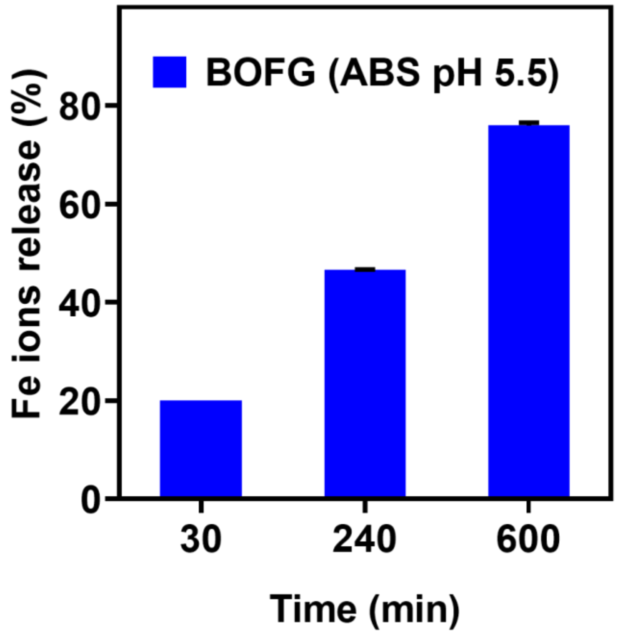


**Figure S4.** Fe ions release from BOFG in ABS (pH 5.5) *via* 1,10-phenanthroline coloration reaction.

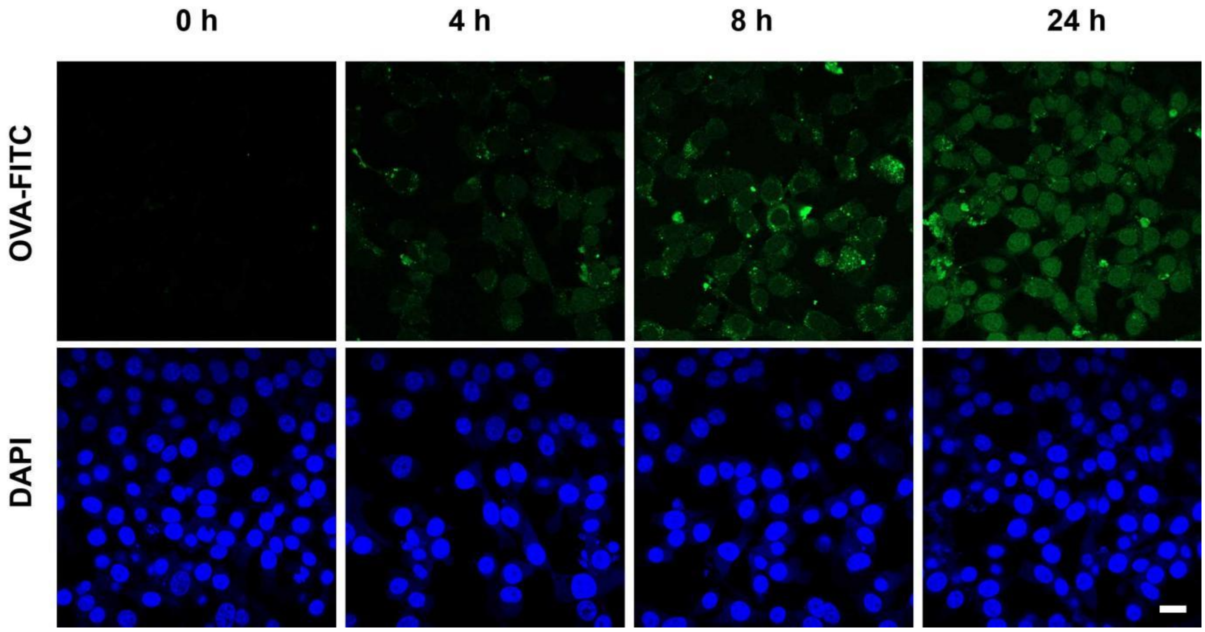


**Figure S5.** Confocal laser scanning microscopy (CLSM) images of CT26 cells after incubation with FITC-labled OVA (BOFG) for different periods. Scale bar = 20 μm.

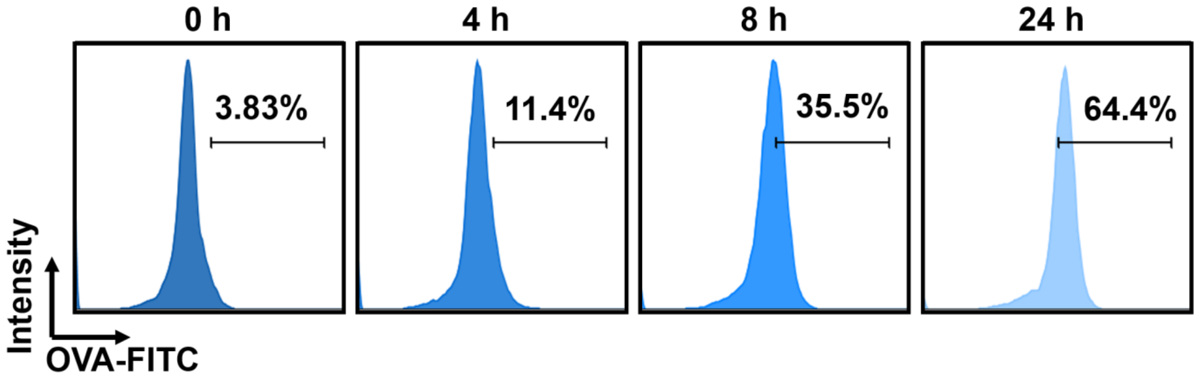


**Figure S6.** Flow cytometry results of CT26 cells after incubation with FITC-labeled OVA (BOFG) for different periods.

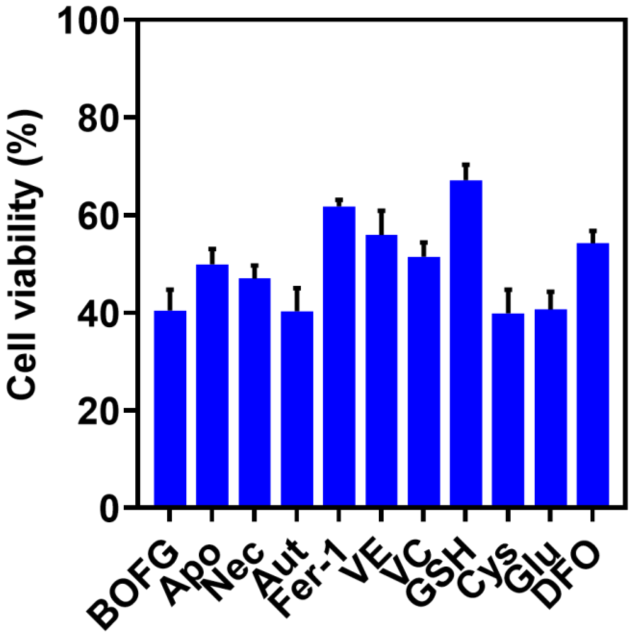


**Figure S7.** Viability of CT26 cells treated with BOFG ± Ac-DEVD-CHO (Apo, 50 µM), Necrostatin-1 (Nec, 490 nM), 3-Methyladenine (Aut, 60 µM), ferrostatin-1 (Fer-1, 100 nM), vitamin E (VE, 20 µM), sodium ascorbate (VC, 20 µM), glutathione (GSH, 1 mM), cystine (Cys, 1 mM), glutamic (Glu, 1 mM), and deferoxamine (DFO, 100 µM).

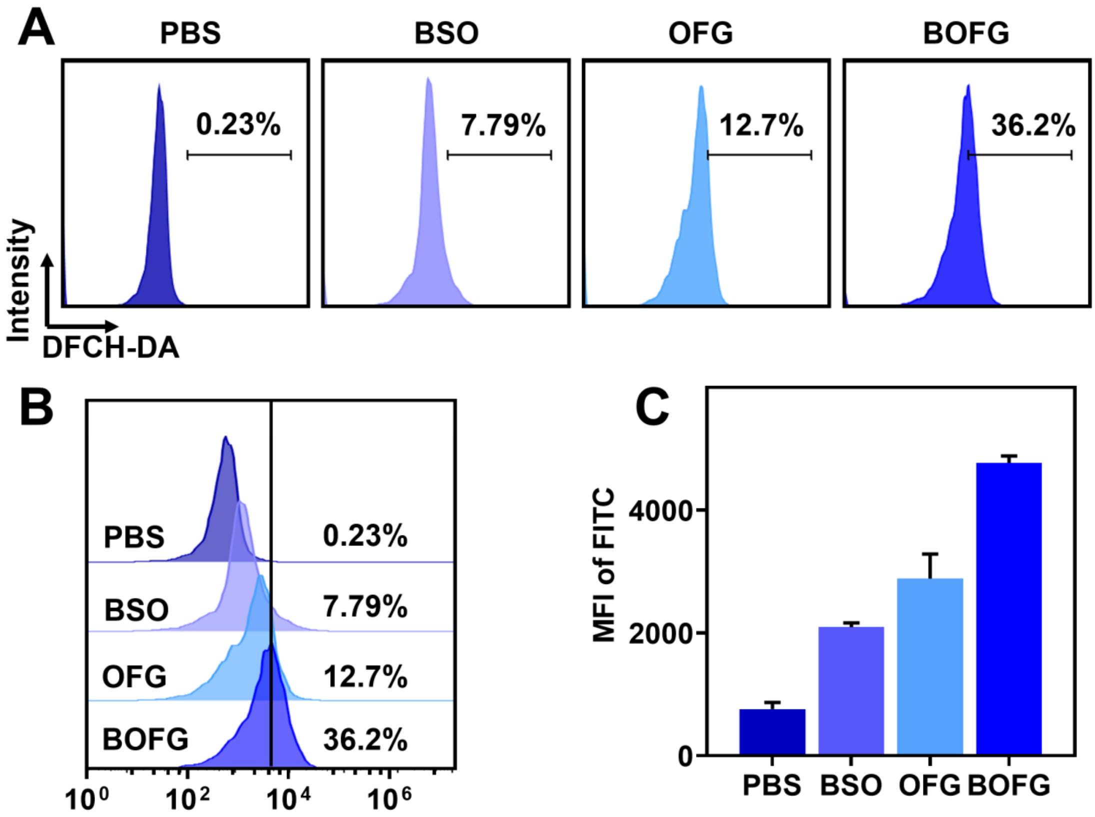


**Figure S8.** A) and B) Flow cytometry results and C) Mean fluorescence of DCFH-DA assay detecting intracellular ROS level of CT26 cells after various treatments.

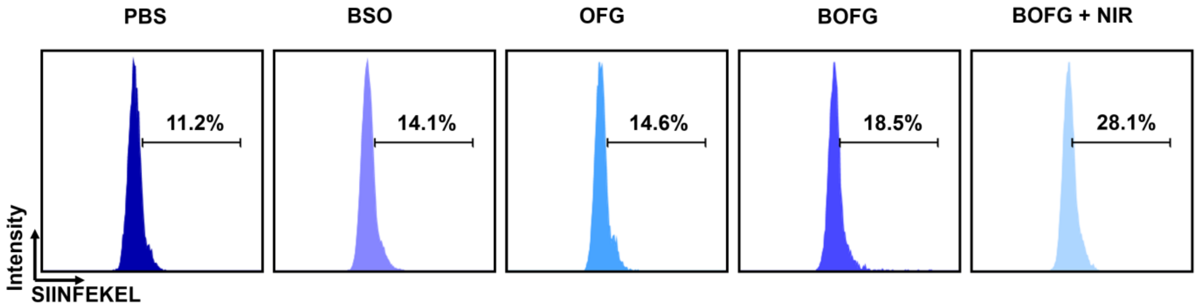


**Figure S9.** The expression level of the MHC-I SIINFEKL complex was measured by flow cytometry.

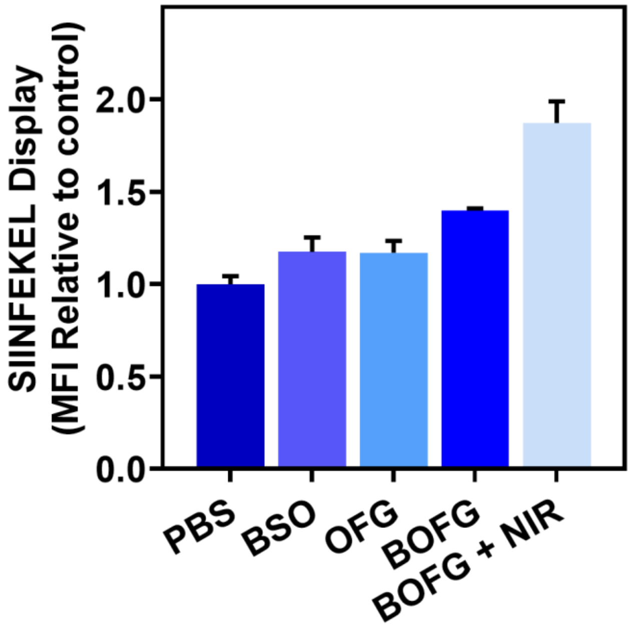


**Figure S10.** Mean fluorescence of the MHC-I SIINFEKL complex (MFI relative to control).

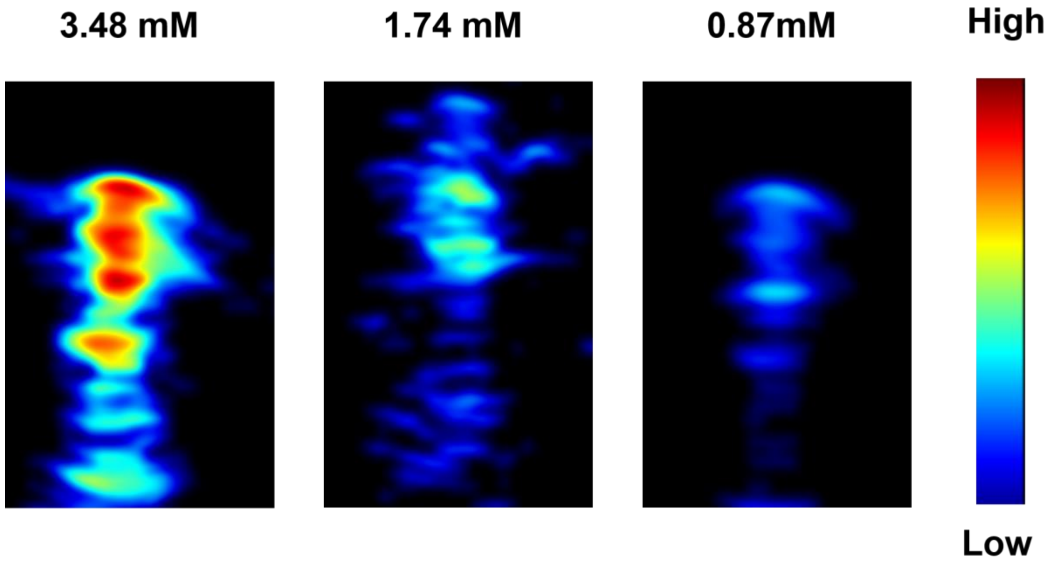


**Figure S11.** *In vitro* PA images of BOFG at different concentrations.

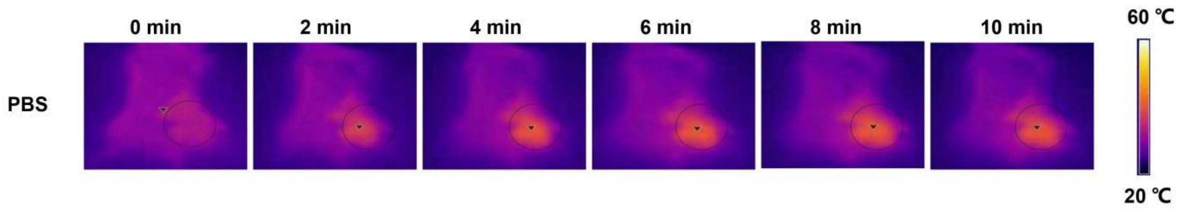


**Figure S12.** Temperature variation of mice at tumor site with laser irradiation at a density of 1 W/cm$^2$ for 10 min, after 8-hour post-injection of PBS.

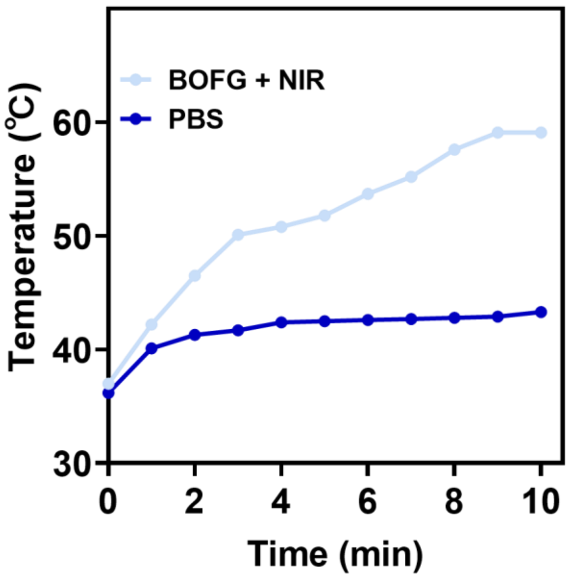


**Figure S13.** Temperature variations of mice at tumor site with laser irradiation at a density of 1 W/cm$^2$ for 10 min, after 8-hour post-injection of PBS and BOFG.

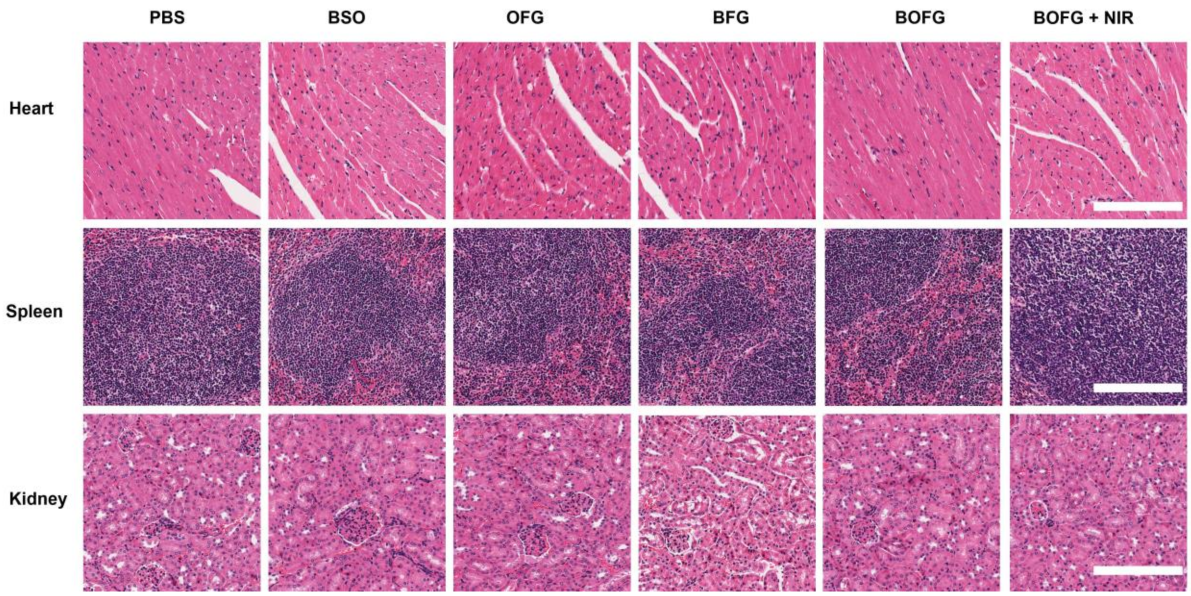


**Figure S14.** H&E staining images of heart, spleen and kidney after various treatments. Scale bar = 200 µm.

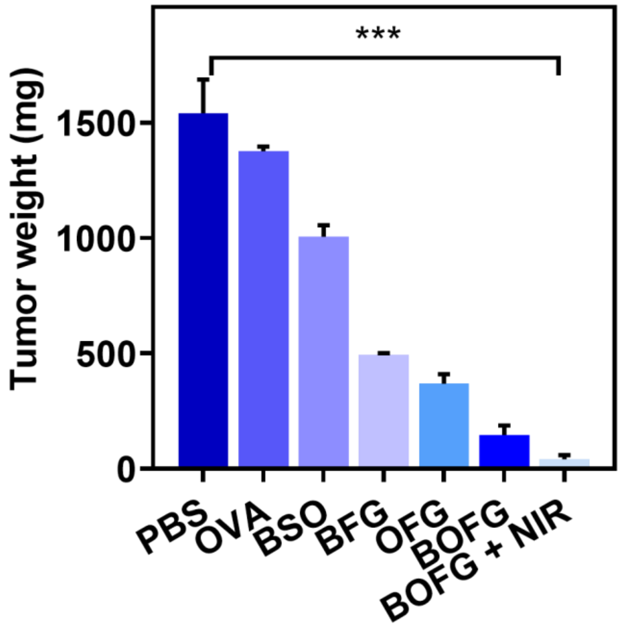


**Figure S15.** Primary tumor weights in mice given different treatments. Data are presented as the mean ± SD (n = 4). n.s. represented no significance, $^{*}p < 0.05$, $^{**}p < 0.01$, $^{***}p < 0.001$.

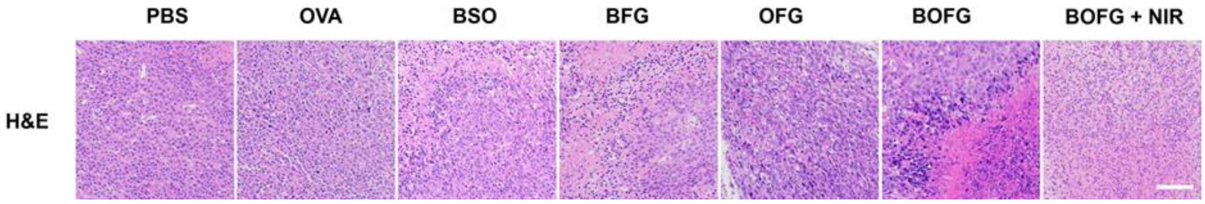


**Figure S16.** H&E staining of primary tumors from different groups. Scale bar = 100 μm.

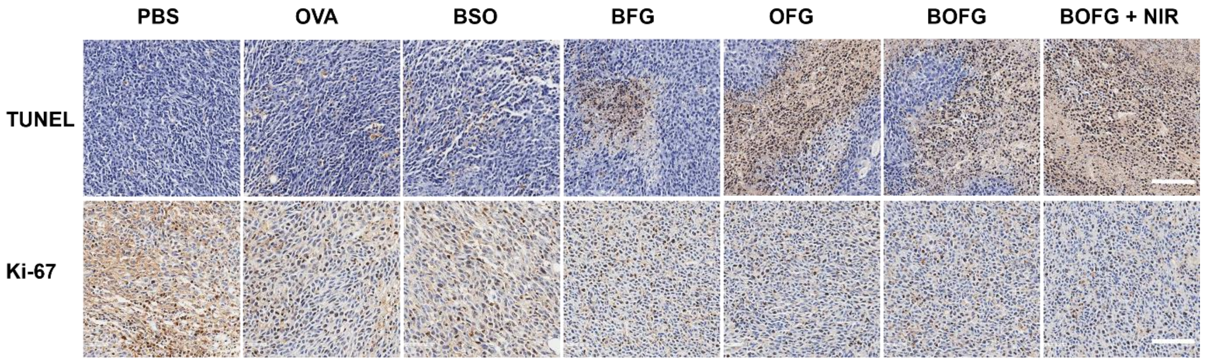


**Figure S17.** TUNEL and Ki-67 staining of primary tumors from different groups. Scale bar = 100 μm.

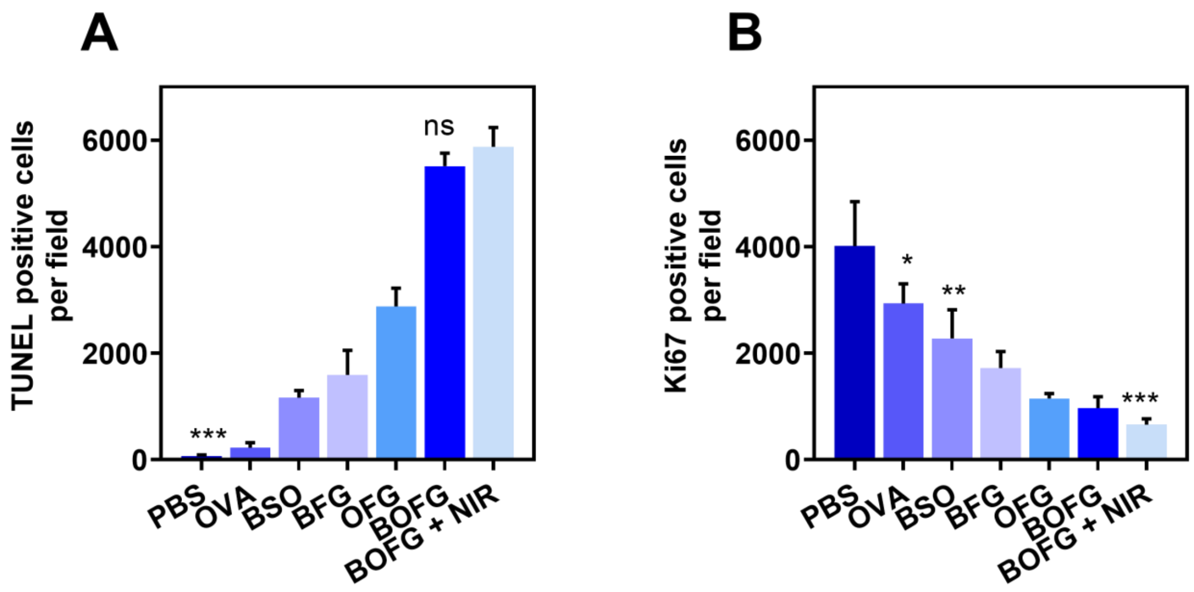


**Figure S18.** A) TUNEL and B) Ki-67 staining of CT26 tumors from different groups. n.s. represented no significance, $*p < 0.05$, $**p < 0.01$, $***p < 0.001$.

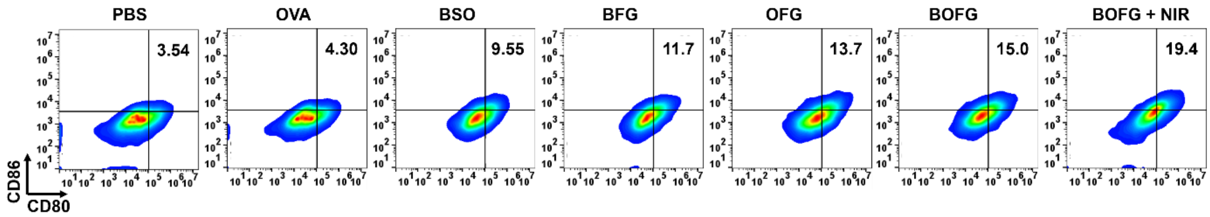


**Figure S19.** A representative flow cytometry analysis displaying the absolute percentage of CD80 and CD86 on the surface of DCs in tumor after different treatments.

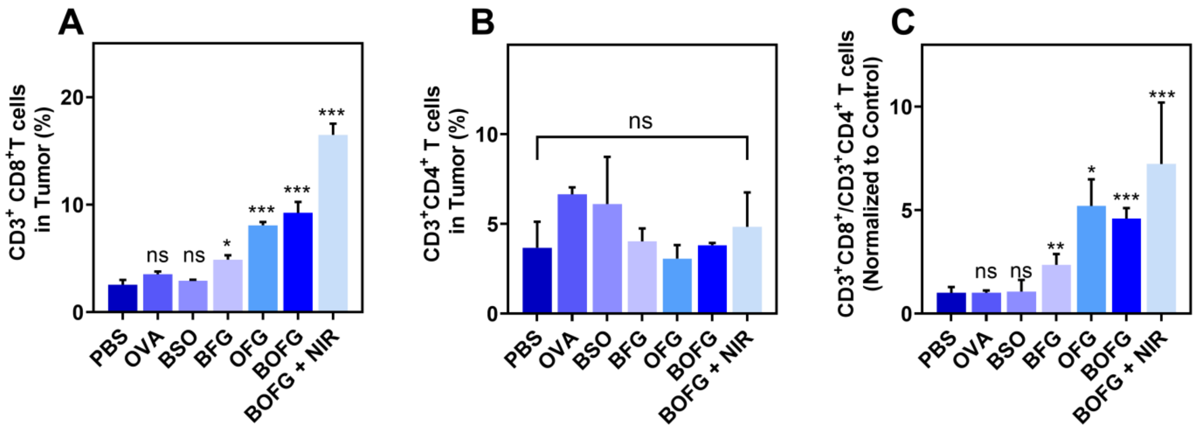


**Figure S20.** The absolute percentages of A) $CD3^+CD8^+$T cells, B) $CD3^+CD4^+$T cells and C) the normalized ratio of $CD3^+CD8^+/CD3^+CD4^+$T cells in tumor after different treatments. n.s. represented no significance, $*p < 0.05$, $**p < 0.01$, $***p < 0.001$.

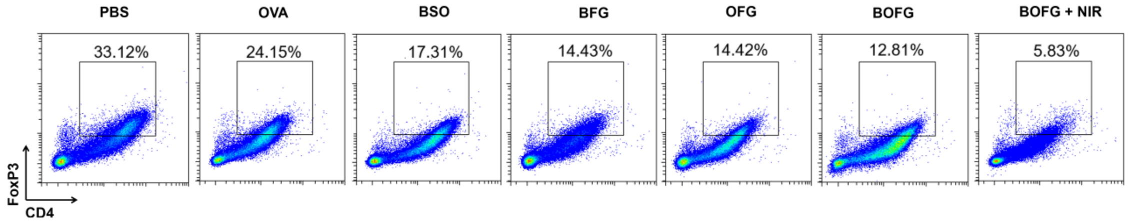


**Figure S21.** A representative flow cytometry analysis displaying the absolute percentage of Foxp3$^+$ T cells and CD4$^+$ T cells in CD25$^+$ tumor cells from mice with different treatments.

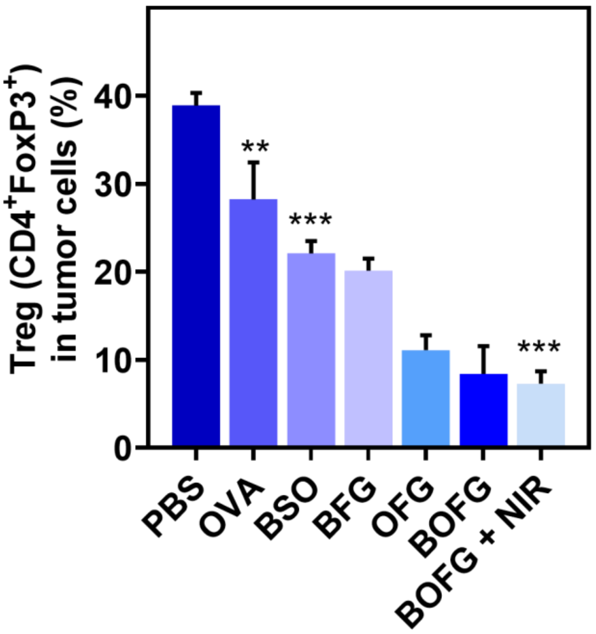


**Figure S22.** The absolute percentages of CD3$^+$CD4$^+$Foxps$^+$Tregs in tumor after different treatments. n.s. represented no significance, *p < 0.05, **p < 0.01, ***p < 0.001.

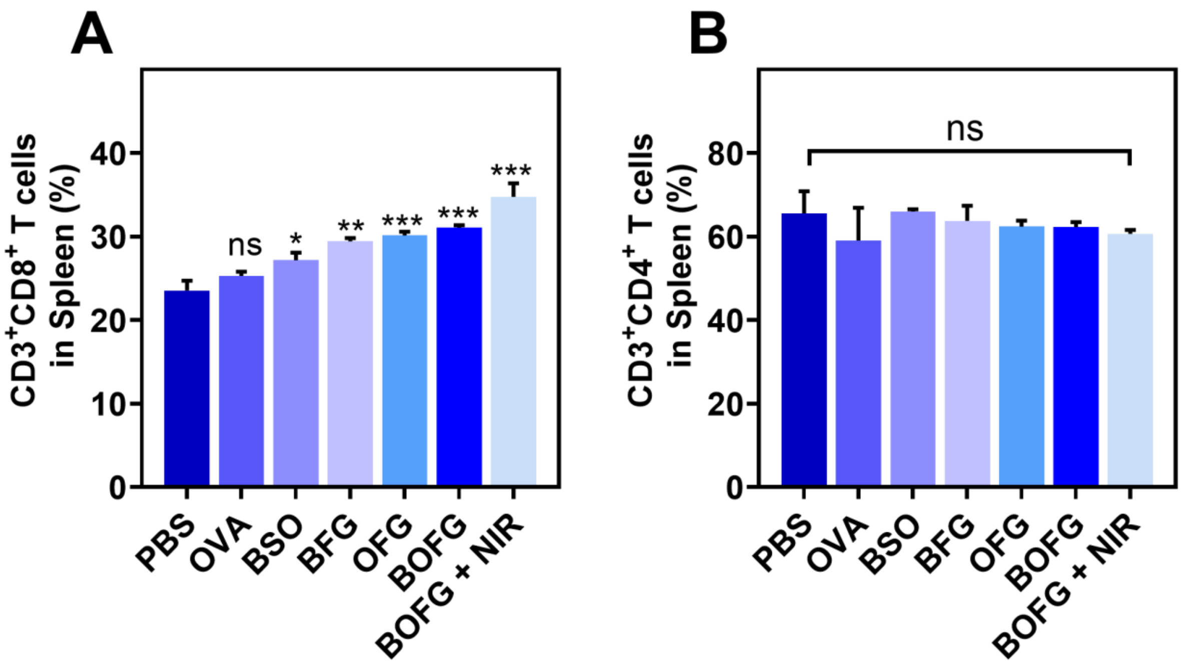


**Figure S23.** The absolute percentages of A) $CD3^+CD8^+$T cells, B) $CD3^+CD4^+$T cells in spleen after different treatments. n.s. represented no significance, *$p < 0.05$, **$p < 0.01$, ***$p < 0.001$.

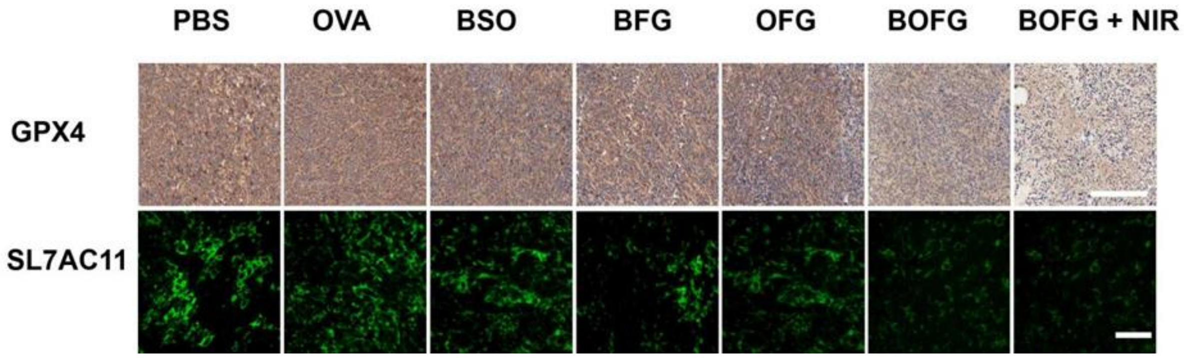


**Figure S24.** GPX4 immunohistochemical staining of primary tumor sections from CT26 cells tumor-bearing mice on the 14th day of treatment. Scale bar =200 μm. Immunofluorescence images of primary tumor slices stained by the anti-SLC7A11 antibody (green). Scale bar = 50 μm.

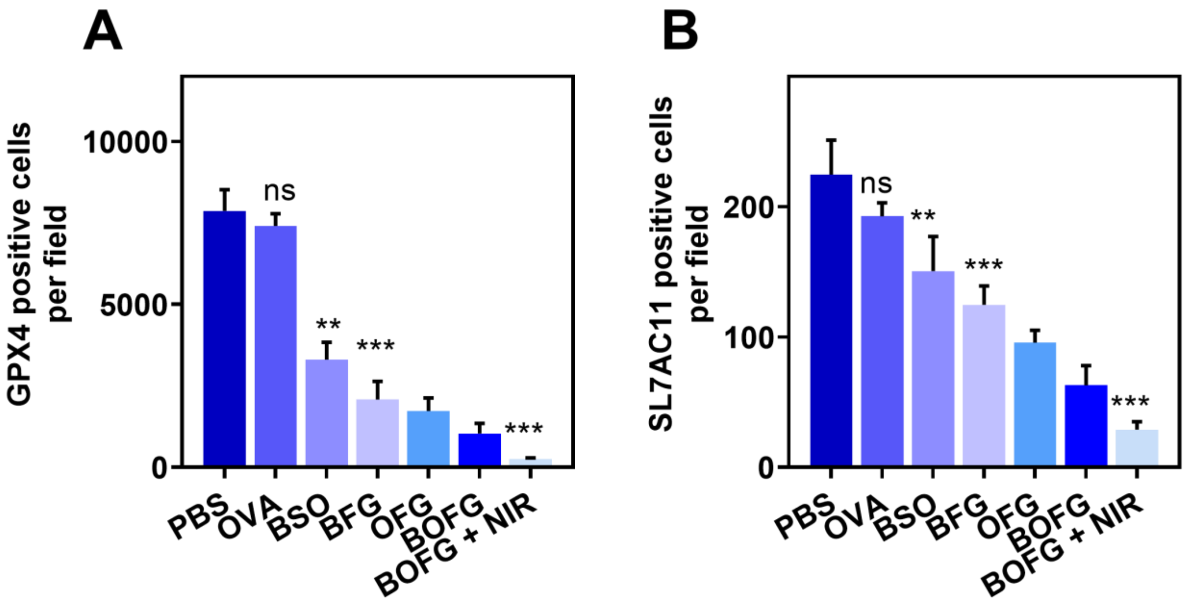


**Figure S25.** GPX4 and SL7AC11 staining of from different groups. n.s. represented no significance, *p < 0.05, **p < 0.01, ***p < 0.001.

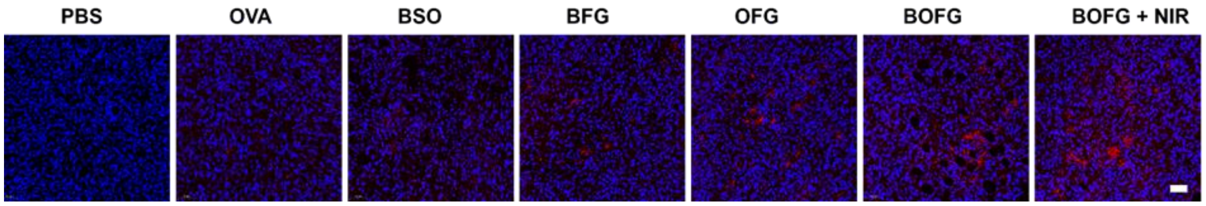


**Figure S26.** Immunofluorescence images of distant tumor slices stained by the anti-IFN-γ antibody (red) and cell nuclei (blue). Scale bar =200 μm.

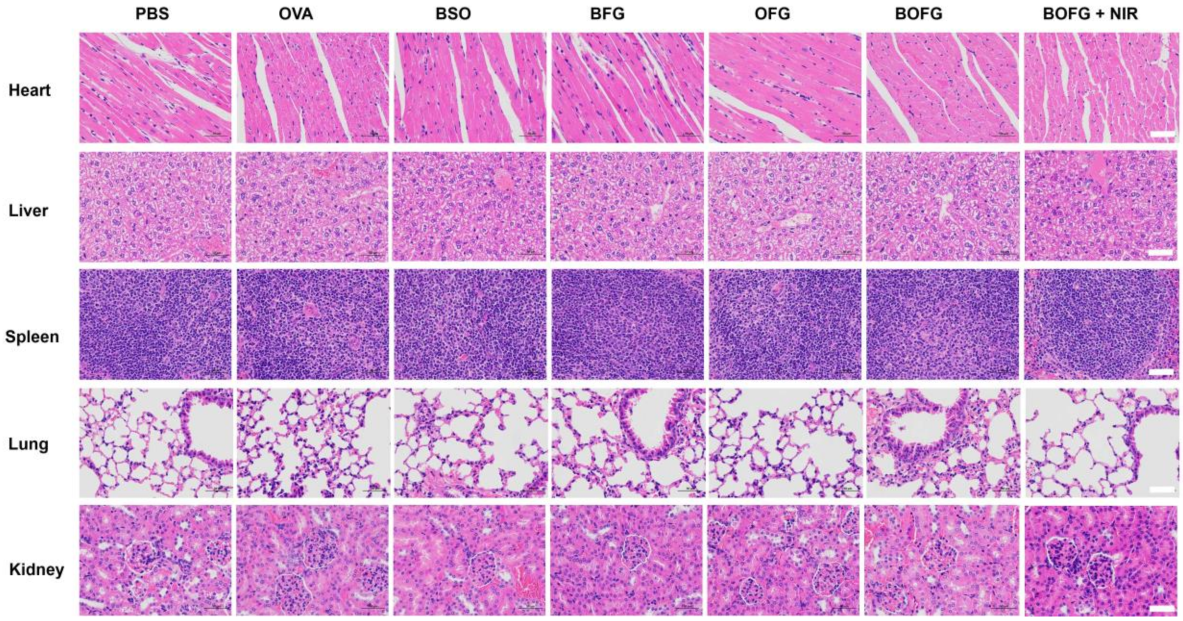


**Figure S27.** H&E staining images of major organs after various treatments. Scale bar =50 μm.

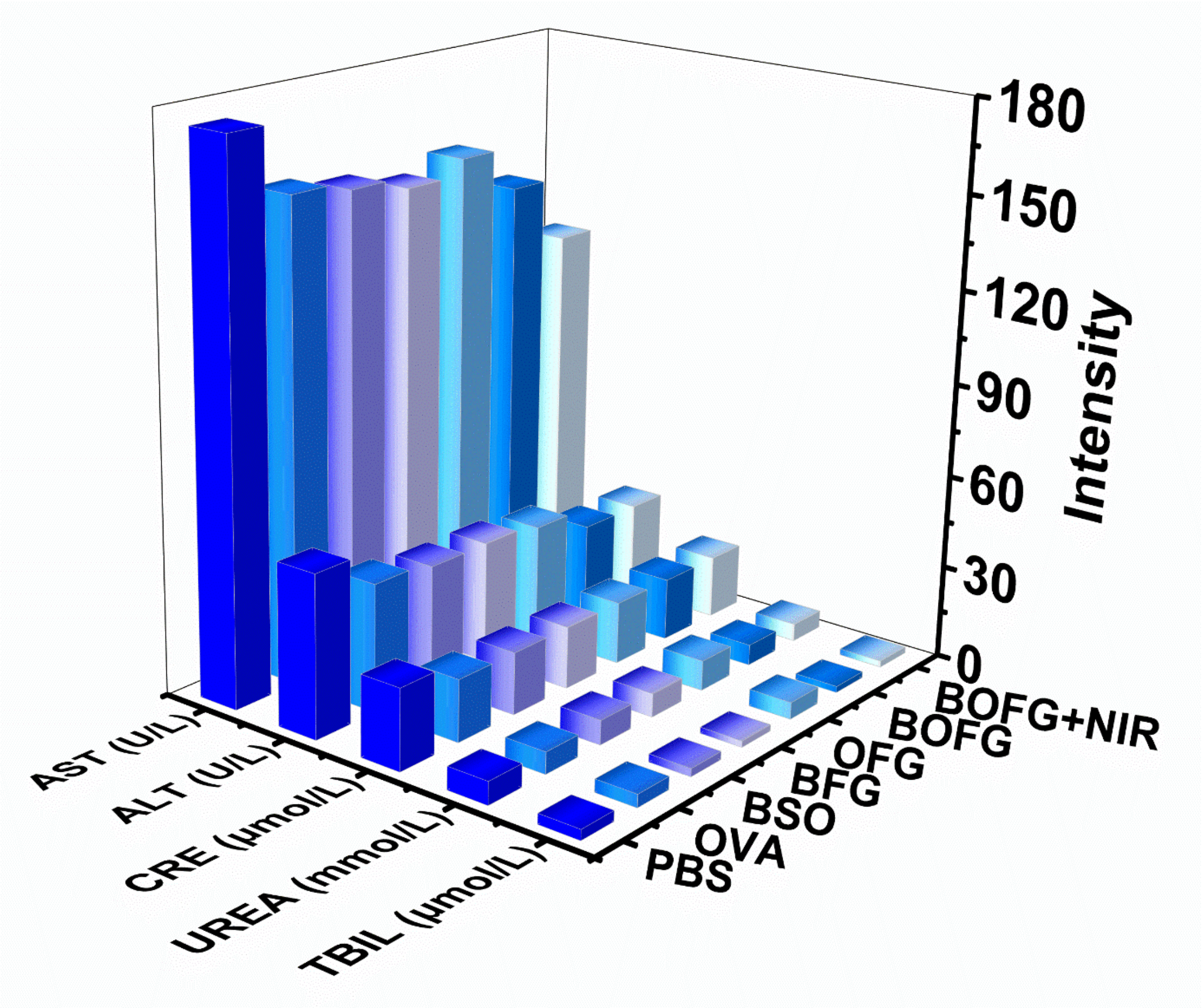


**Figure S28.** Blood biochemistry analysis of the mice after different treatments.

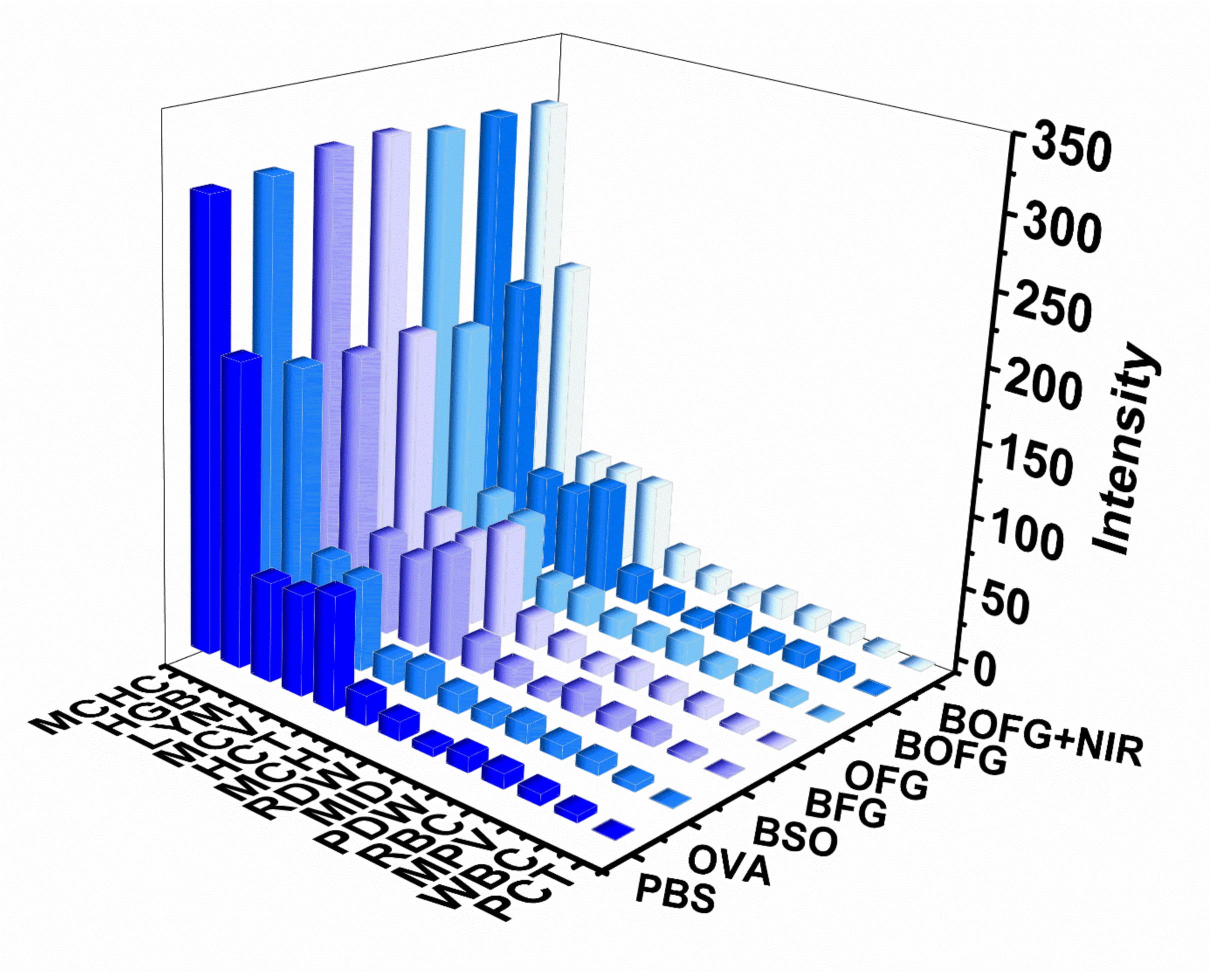


**Figure S29.** Blood routine test of the mice after various treatments.
